# Mechanism of activation and autophosphorylation of a histidine kinase

**DOI:** 10.1038/s42004-024-01272-6

**Published:** 2024-09-03

**Authors:** Mayukh Kansari, Fathia Idiris, Hendrik Szurmant, Tomáš Kubař, Alexander Schug

**Affiliations:** 1 Institute of Physical Chemistry, Karlsruhe Institute of Technology, Karlsruhe, Germany; 2 Steinbuch Centre for Computing, Karlsruhe Institute of Technology, Karlsruhe, Germany; 3 College of Osteopathic Medicine of the Pacific, Western University of Health Sciences, Pomona, CA USA; 4 Jülich Supercomputing Centre, Forschungszentrum Jülich, Jülich, Germany; 5 Faculty of Biology, University of Duisburg/Essen, Essen, Germany

**Keywords:** Kinases, Density functional theory, Molecular dynamics, Enzyme mechanisms

## Abstract

Histidine kinases (HK) are one of the main prokaryotic signaling systems. Two structurally conserved catalytic domains inside the HK enable autokinase, phosphotransfer, and phosphatase activities. Here, we focus on a detailed mechanistic understanding of the functional cycle of the WalK HK by a multi-scale simulation approach, consisting of classical as well as hybrid QM/MM molecular dynamics simulation. Strikingly, a conformational transition induced solely in DHp leads to the correct activated conformation in CA crucial for autophosphorylation. This finding explains how variable sensor domains induce the transition from inactive to active state. The subsequent autophosphorylation inside DHp proceeds via a penta-coordinated transition state to a protonated phosphohistidine intermediate. This intermediate is consequently deprotonated by a suitable nearby base. The reaction energetics are controlled by the final proton acceptor and presence of a magnesium cation. The slow rates of the process result from the high energy barrier of the conformational transition between inactive and active states. The phosphorylation step exhibits a lower barrier and down-the-hill energetics. Thus, our work suggests a detailed mechanistic model for HK autophosphorylation.

## Abstract (web summary)

Histidine kinases (HK) are the main component of a wide-spread signal transduction system in bacteria that are essential for cell viability, however, the details of HK autophosphorylation remain poorly understood. Here, the authors utilize a multi-scale simulation approach to investigate the mechanism of activation and autophosphorylation process, revealing the rate determining step and reaction free energy of the process.

## Introduction

**Fig. 1:**
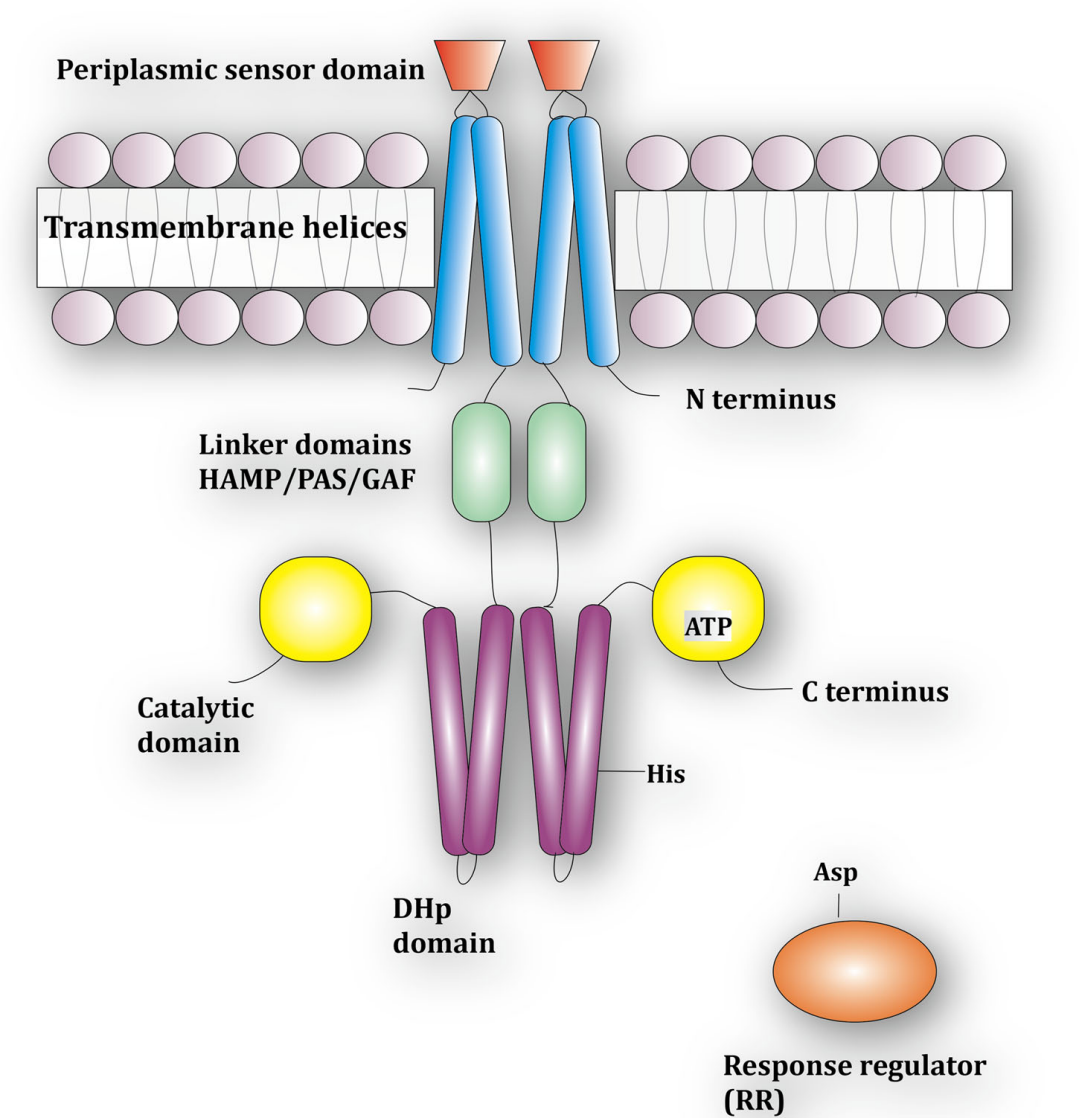
A typical two-component system (TCS) featuring domains for signal recognition, transmission, and catalysis. A stimulus is first detected at the periplasmic sensor domain (red) and this signal is transmitted along the transmembrane helices (blue) and the linker domains (green) before reaching the catalytic core at the C-terminus. The catalytic core comprises the dimerization and histidine phosphotransfer (DHp, purple) and catalytic ATP-binding (CA, yellow) domains. Signal detection results in a phosphoryl transfer reaction from ATP in the CA domain to a conserved histidine in the DHp domain. This phosphoryl group is then transferred to an aspartic acid in the response regulator (orange) protein, resulting in an appropriate cellular response. A labeled cartoon model can be found in Fig. 4.

Two component systems (TCS) are one of the major signal transduction mechanisms in bacteria. They regulate the response to a variety of environmental or cellular signals (temperature changes, changes in pH, ligands, etc.)^1^. The individual components are the sensor histidine kinase (HK) that detects the signal and the response regulator (RR) protein that coordinates the response, most commonly by acting as a transcription factor (see Fig. 1). The two proteins communicate via histidine to aspartate phosphoryl-group transfer. Based on domain architectures, evolutionary origin and activities there are numerous variations of TCS^2,3^. While TCS are employed by some eukaryotes, they are notably absent from the animal kingdom. That, paired with their importance to bacteria makes these enzymes promising targets for developing novel compounds that selectively inhibit the growth of bacteria or suppress virulence. For instance, waldiomycin, an angucycline antibiotic, inhibits the HK activity of WalK^4,5^ in *Staphylococcus aureus*, a human pathogen responsible for a variety of acute and chronic diseases^6–8^. The molecular signal of this system is still unknown but emanates from the bacterial cell wall^9^. In general, the WalRK system has garnered significant experimental attention since it is conserved across Gram-positive bacteria of the order *Firmicutes* where it has been shown to be essential for viability in a variety of different species of bacteria. Due to their prevalence and the associated wealth of genomic data, TCS are also a common target of bioinformatics studies to, e.g., investigate TCS complex formation^10^, predict^11^ or investigate^12^ conformational transitions, or redesign protein signaling^13^. Other extensive studies and reviews highlight the range of TCSs and their activities^14–18^.

**Fig. 2:**
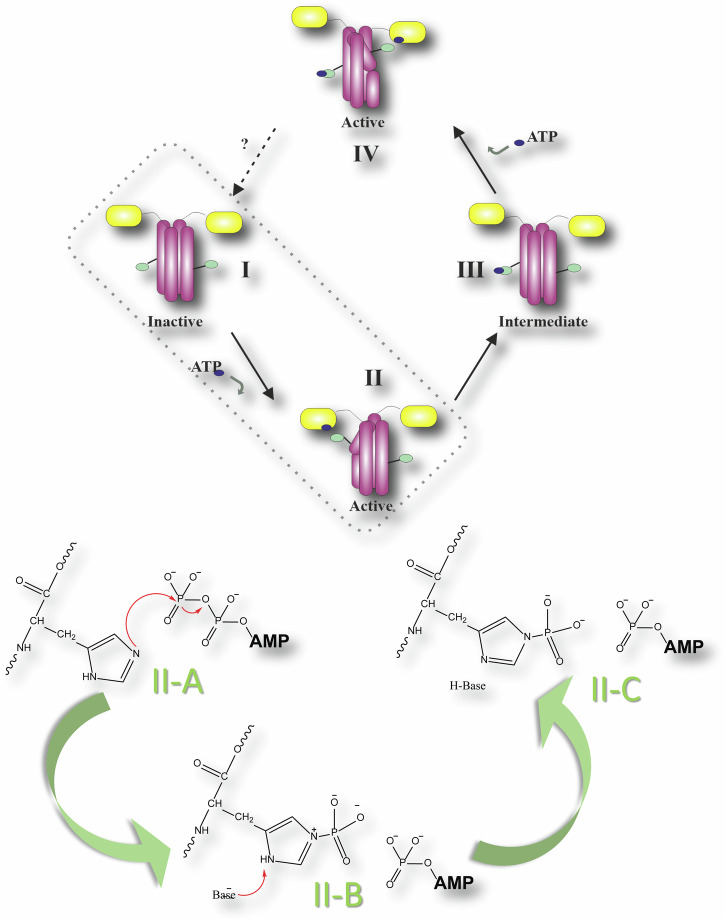
Two different processes in an HK are taking place on different time scales. Purple – DHp domain; yellow – catalytic domain; black dot – phosphate group. This work aims to investigate the processes I → II (classical atomistic simulation) as well as the phosphorylation in state II (QM/MM simulation). The chemical reaction consists of two distinct steps: II-A → II-B, phosphoryl transfer itself, and II-B → II-C, deprotonation of the phosphorylated histidine.

In a prototypical TCS (cf. Fig. 1), a stimulus (environmental or cellular signal) is detected by the periplasmic and/or cytoplasmic sensor domains at the N-terminus of the dimeric HK. This signal is propagated from N- to C-terminus along the multidomain HK via a series of conformational transitions ruled by unstable transient interactions^19^. While the structural properties of HKs differ, they all have at the C-terminus a conserved kinase core (~450 amino acids) consisting of the homodimeric dimerization histidine phosphotransfer (DHp) domain and the ATP-binding catalytic domain (CA). Following signal detection, the conserved core adopts an asymmetric conformation such that one of the two subunits of the homodimer is kinase active while the other is inactive (cf. Fig. 2). In the kinase inactive conformation, ATP can enter the CA domain and the binding site of the DHp domain is accessible to a RR for phosphoryl-group transfer. In the kinase active conformation, the RR cannot bind the DHp domain. Here, the gamma-phosphoryl group of the bound ATP of one CA is positioned in close proximity to a specific phosphorylatable histidine of DHp. Two different auto-phosphorylation mechanisms have been attributed to individual HKs: In cis-phosphorylation the ATP from the CA domain phosphorylates its own DHp domain within the homodimer, while in trans-phosphorylation the DHp domain on the other monomer within the homodimer is phosphorylated^20^. It appears that the difference in phosphorylation mechanism is merely structural, based on a left handed versus right handed orientation of the dimeric four-helix bundle that forms the DHp domain. As soon as the histidine is phosphorylated, transfer of this phosphoryl group to an aspartate of a bound RR for communication between the two proteins is possible. Bifunctional HK (e.g., EnvZ) also function as phosphatase for the RR and therefore catalyse the hydrolysis of the phosphoryl group^21^. The activation and inactivation mechanisms of the protein are reviewed in detail in ref. ^22^.

In this study, the activation mechanism and subsequent autophosphorylation reaction of the WalK HK [also known as YycG^15^] are explored. WalK is an essential HK involved in the regulation of cell growth and division^23^ in low GC-content Gram-positive bacteria such as *Bacillus subtilis*^24^ and *S. aureus*^25^. Building on the tremendous insights into HK function already gained from structural investigations, we focus on several key-aspects of HK autophosphorylation that remain only partially or poorly understood. To complete our understanding of the autophosphorylation mechanisms we utilize a complex multi-scale computational study on supercomputers to complement previous experimental findings.

First, we aim to understand the conformational dynamics and free energy barrier of the transition from inactive to active conformation. While the initial and final structures of the transition have been well resolved in X-ray experiments, insight into conformational dynamics – experimentally poorly accessible – can be provided by molecular dynamics (MD) simulations. Pending on the task, we employ both coarse-grained and regular simulations. Another critical gap in our understanding of the signal transduction event is how signal detection by the N-terminal signal domains induce the conformational transition by the C-terminal catalytic core that leads to activation. We address this gap of knowledge by mimicking effective forces from an incoming signal on the DHp domain and show that these signaling forces trigger the conformational transition of the entire HK. Finally, we complete the picture of HK activation by investigating the mechanism and the energetics of the histidine phosphorylation reaction. This histidine phosphorylation requires an acidic residue near the target histidine^26–29^. We resolve the sequence of elementary events of the phosphorylation and the reaction energetics by extended-sampling QM/MM simulations on a microsecond scale^30^ to distinguish the order of phosphoryl transfer and proton transfer and the influence of a critical magnesium cation^28,31,32^. Taken together, the combination of results for the conformational transition and the chemical phosphorylation step renders the course of energy of the beginning of the TCS phosphorylation cascade, thereby revealing the rate determining step and providing insights on the free energy that is released during the phosphoryl transfer.

## Methods

A detailed description of the simulation methods is provided supplementary information.

### Molecular dynamics (MD) simulations

The inactive and active states of histidine kinase WalK, an essential HK from *Lactobacillus plantarum*, were obtained from the protein data bank (PDB ID: 4U7N and 4U7O respectively)^33^. The non-terminal missing residues were modelled using MODELLER version 10.0^34^. The phosphoryl acceptor His391 residue was prepared in the HID state, so that the deprotonated N*ϵ* atom is a suitable phosphorylation target, and there is a strong hydrogen bridge between the protonated N*δ*–H and the Glu392 sidechain, which also supports a proton transfer between pHis391 and Glu392. The terminal Glutamate is negatively charged at physiological pH. All MD simulations of WalK HK were conducted at 300 K using GROMACS 2020.4^35–37^ patched with PLUMED v2.7^38^, with AMBER99SB-ILDN force-field^39^, and with a time step of 2 fs. Each HK state was solvated with the TIP3P water model^40^. Electrostatic interactions were calculated using the particle–mesh Ewald (PME) method^41^ with a direct cutoff of 1.0 nm, and a cutoff of 1.0 nm was used for the van der Waals interactions. All bonds involving hydrogen were constrained by the LINCS algorithm^42^. Each HK-water system was first minimized using the steepest descent algorithm for 1000 steps. Then, NVT equilibration was performed for 1 ns using the velocity-rescaling thermostat^43^, allowing the system to reach temperature convergence. Once the temperature was stabilized, NPT equilibration was carried out for 1 ns with the Parrinello–Rahman barostat^44^, effectively achieving pressure convergence. The 2D metadynamics simulations used two different collective variables (CVs): the difference of root-mean-squared deviations from the inactive and active structures (PDB ID: 4U7N and 4U7O, respectively) termed “ΔRMSD”, and a torsional angle defined by the centers of mass (COM) of four different parts of the protein: COM1: chain A residues 454–610, COM2: chain A residues 389-453, COM3: chain B residues 454–610, COM4: chain B residues 389–453. The torsional angle COM1–COM2–COM3–COM4 is then termed “COMTOR”.

### Inactive to active state conformational transition

To study the large-scale conformational changes involved in the activation pathway of the WalK HK, we used steered MD simulation, and then metadynamics simulations to obtain the free energy profile. We chose these methods since the endpoint conformations of the WalK autophosphorylation reaction have been experimentally determined. In the steered MD we used the difference in RMSD from the two reference states as the collective variable to obtain the intermediate configurations along the transition pathway. The RMSD bias was applied to C*α* atoms of the DHp and the active CA domain as detailed in Materials and Methods. Finally, a converged free energy landscape of the conformational transition was obtained by performing a multiple-walker metadynamics simulation with two collective variables, *Δ*RMSD and additionally, an inter-domain torsional angle COMTOR, to facilitate convergence.

### Hybrid QM/MM metadynamics simulations

All QM/MM simulations were performed using a combination of locally modified versions^45–47^ of Gromacs^35,36^ and DFTB+^48^ interfaced with Plumed^49^. The initial structural model is based on the crystal structure PDB ID 4U7O^33^; the details of the initial modelling can be referred to in the SI. The MM region was treated with the AMBER99SB-ILDN force field for the protein^39^ and TIP3P water model^40^. The QM region, defined in the Results section, was described with the DFTB3 method^50^ using the 3OB parameter set^51^. Special DFTB repulsive potentials were used for the interactions P–N^52^ and P–O^53^, specifically calibrated to properly describe the phosphoryl transfer and hydrolysis reactions. The electrostatic interactions between the QM and MM regions were treated by means of electronic embedding, and all of the QM–MM interactions were evaluated with PME, thus no long-range cut-off was applied on the electrostatics. A constant temperature of 300 K was maintained by means of Bussi’s thermostat^43^, and the simulations ran at constant volume. PMFs were generated using the multiple walker well-tempered metadynamics protocol^54,55^ involving 96 individual QM/MM simulations ("walkers”). The convergence of the PMFs was monitored, and the metadynamics sampling was extended to 1 *μ*s in each of the two cases.

## Results

### The conformational transition is endergonic but creates new favorable inter-domain contacts

The steered MD showed that the catalytic ATP-binding domain gradually rotated by 57 °, and the center-of-mass (COM) distance between the DHp and CA domains decreased from approximately 2.55 to 1.92 nm. This brings the phosphorylatable His391 in the DHp domain closer to the ATP-lid (Leu545–Leu577) in the CA domain to form the asymmetrical active state. It should be noted that ATP molecules were not present in these simulations and thus ATP interactions with the HK cannot be quantified and characterized by these simulations. The making and breaking of the inter-domain contacts will be reported in more detail below.

**Fig. 3:**
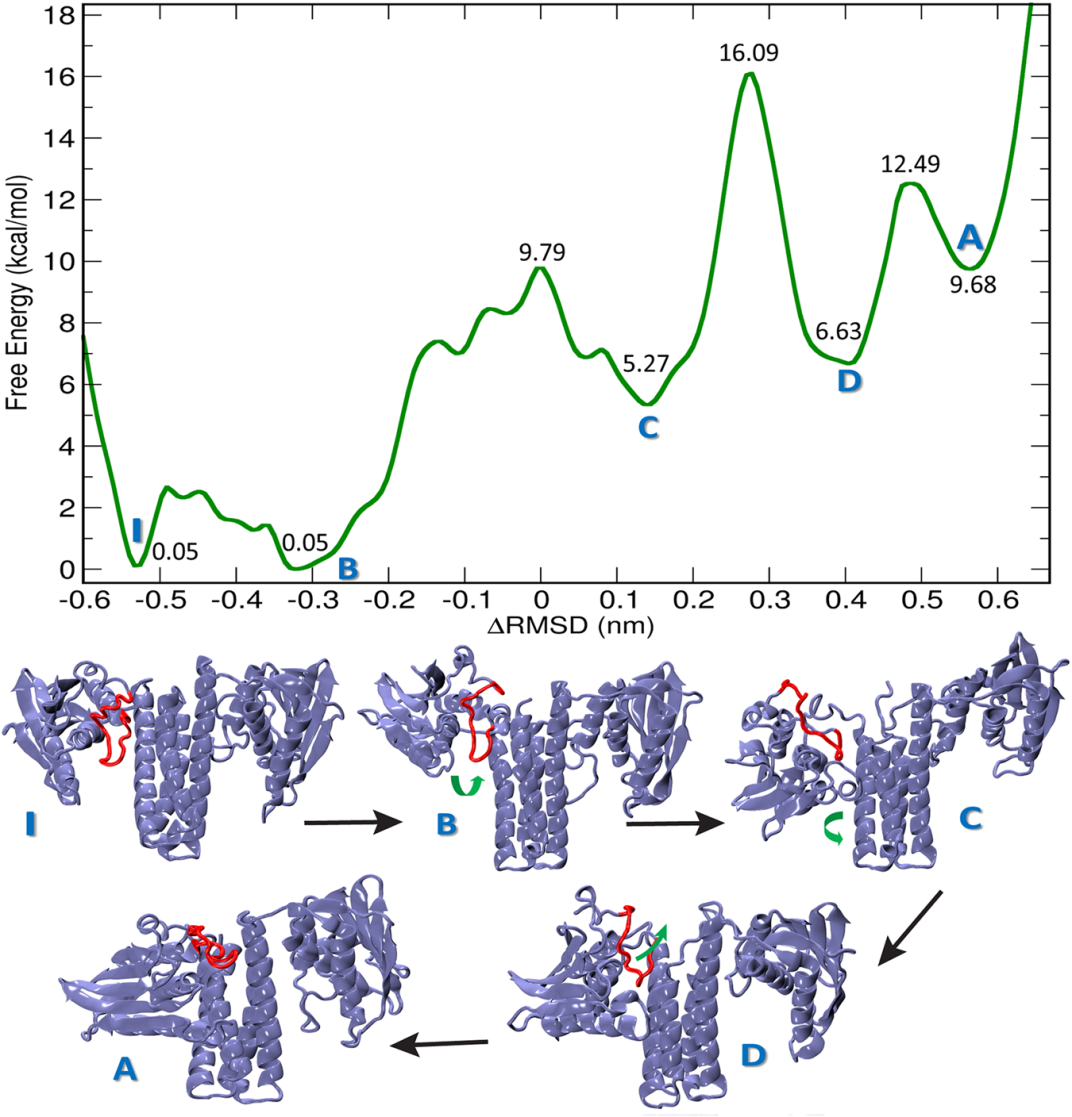
Transition between inactive and active structures via intermediates. Top: The free energy profile of the conformational transition obtained from the 2D metadynamics simulation as a function of ΔRMSD, with COMTOR integrated out. Bottom: Representative structures of the conformational states found on the free energy profile, obtained as centroids from cluster analyses of additional unrestrained MD simulations started from geometries found in the metadynamics simulation. I—inactive structure; B, C, D—intermediates; A—active structures. The loop region Arg558--Thr573 is highlighted in red. Green arrows are pointing at the large-scale movements observed.

The free energy surface resulting from the 2D-MTD simulations is shown in Fig. 3 as a function of *Δ*RMSD with COMTOR integrated out, while the original 2D free energy surface may be referred to in Fig. S1. Convergence of the Free Energy profile (FEP) for this simulation is shown in Fig. S12. The conformational transition barrier (state C to State D) (rate determining step) remains unchanged. The initial, inactive conformation ‘I’ is followed by a closely similar conformation ‘B’ with the same energy. Then, a barrier of 10 kcal/mol has to be overcome at ΔRMSD ≈ 0 nm. That barrier is largely due to the rotation of the CA domain, leading to the intermediate conformation ‘C’ with an introduction of asymmetry, and lies 6 kcal/mol higher than the initial, inactive state. Note that one of the protein monomers is now beginning the transition into the active conformation, while the other monomer will remain in the inactive conformation throughout our investigation. After that appears the rate-limiting barrier of 16 kcal/mol, which is due to the translation of the CA domain towards the DHp domain, leading to another intermediate conformation ‘D’. The final product ‘A’ lying +10 kcal/mol in free energy higher than the reactant I is reached after passing another, minor barrier. The transition from the state D to A consists mostly of the movement of the loop region Arg558–Thr573 from the interface between the CA and DHp domains, flanking outwards, accompanied by a further approach of the ATP-lid to the phosphorylatable His391 situated in DHp domain. It is in this step that the ATP-lid finally assumes the conformation needed to bind ATP, and consequently, carry out the autophosphorylation reaction. Interestingly, the loop region Arg558–Thr573 is more flexible in the A state than in all of the states B, C and D, as can be inferred from the corresponding Ramachandran plots in Figs. S5–S8.

To characterize the conformational changes during the activation of WalK, we analyzed the formation and breaking of contacts between the different parts of the CA and DHp domains. First, let us turn our attention to any inter-domain salt-brige interactions between the CA and DHp domains. Interestingly, the number of salt bridges increases along the entire process: While the inactive states I and B possess two and one salt bridges, respectively, three salt bridges are observed in each of the intermediate states C and D, before as many as six salt-bridge interactions are identified in the active state A. In addition, there is a new salt bridge between Asp563 and the residue Arg453 on the other WalK monomer in the active state, providing an extra stabilization to the previously mentioned loop region in the active site, thus creating a suitable ATP binding site. All the salt-bridge interactions of the active conformer are shown in Fig. S2.

**Fig. 4:**
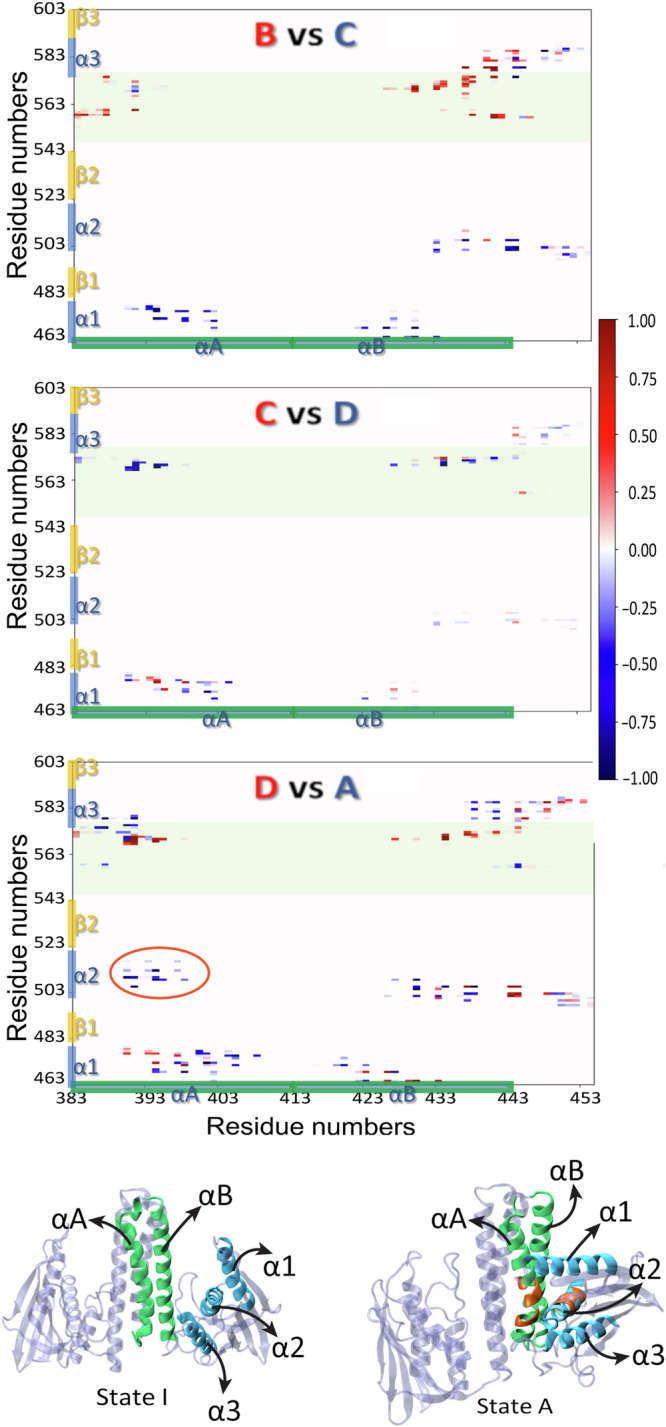
Concerted activation pathway of histidine kinase. Top: Inter-domain differential contact frequency maps for the interface between the DHp and CA domains. The residues in the DHp domain are on the horizontal axis, and the CA domain is on the vertical, with the ATP-lid highlighted in green. The three different plots are separate maps for each of the three consecutive conformational transitions occurring during the activation process: B → C, C → D and D → A, respectively. Contacts are defined as non-bonded interactions between heavy atoms within a cut-off distance of 0.45 nm. The color-coded values range between—1 (for contacts present entirely in the initial state and missing entirely in the final state) and  + 1 (for contacts missing entirely in the initial state and present entirely in the final state). Bottom: Representative structures of the states I and A. The two DHp domains of the homodimer form the central hour-helical bundle, the two catalytic domains are connected by a linker region on the left and right of the helical bundle. Highlighted are the *α*-helical segments of one monomer: *α*1 (residues 460–476), *α*2 (502–520) and *α*3 (575–588) in blue; *α*A (383–413) and *α*B (415–445) in green. Contacts that only occur in the active state A are encircled in the contact maps, and highlighted in orange in the structure of state A.

To describe the interaction between the different parts of CA and DHp domains more generally, we have estimated the formation and breaking of general contacts during the transitions B → C, C → D and D → A. In order to do so, we ran additional unrestrained MD simulations starting from geometries taken from the metadynamics simulation, belonging to each of the states B, C, D and A, and estimated the frequencies of contacts between the individual amino acid residues. These data were used to generate the corresponding differential contact frequency maps, which are shown in Fig. 4.

Four regions in the difference contact frequency maps, where contact formation is favorable, have been identified. The number of CA–DHp contacts between residues Thr395–Gly410 of the DHp and residues Lys475–Pro480 in the CA *α*-helix increased significantly in the B → C step. So did the contacts between DHp residues Leu422–Asp430 and CA residues Asn460–Lys476, with some reordering observed in the D → A step. For example, the interaction between Val424–Lys476 broke in order for Val424–Arg469, Val424–Met472 and Ser402–Lys476 interactions to form. A further region of interest is between DHp residues Glu392–Ser402 and the CA loop region Phe559–Ile579. Contact pair formation in this region became increasingly more favorable as the two domains were brought closer together in steps C → D → A. Finally, the contact between the *α*-helical regions 391–399 (*α*A) and 504–517 (*α*2) comes into being in the step D → A, and is only found in the active state A.

Finally, any contacts between hydrophobic and aromatic residues were analyzed specifically in the interface region of DHp and CA domains. This was performed by restricting the procedure for creating differential contact maps, to the residues of Val, Phe, Ile, Met, Leu and Tyr. The resulting contact maps shown in Fig. S4 reveal that there are no significant changes of hydrophobic/aromatic contacts between these regions.

### Helical bending and catalytic ATP-binding domain rotation occurs concurrently

We next aimed to explain the relationship between the motions of the DHp and CA domains. Above, we had explored a scenario in which HK activation conformation transition occurs via a concerted mechanism as the biasing *Δ*RMSD potential was applied to both domains. In this approach, the helical bending of the DHp and the rotation of the CA domain necessary for activation occurs simultaneously. In a full-length protein, transition forces stem from N-terminal signal binding domains that are directly tethered to the DHp but not the C-terminal CA domain. We thus investigated an alternative approach where the domains move independently step-wise. The RMSD biasing potential in the steered MD simulation was only applied to the C*α* atoms of the DHp domain (cf. Methods). This means that the CA domain was left unperturbed and the structural transition was only induced on DHp by the local perturbation.

**Fig. 5:**
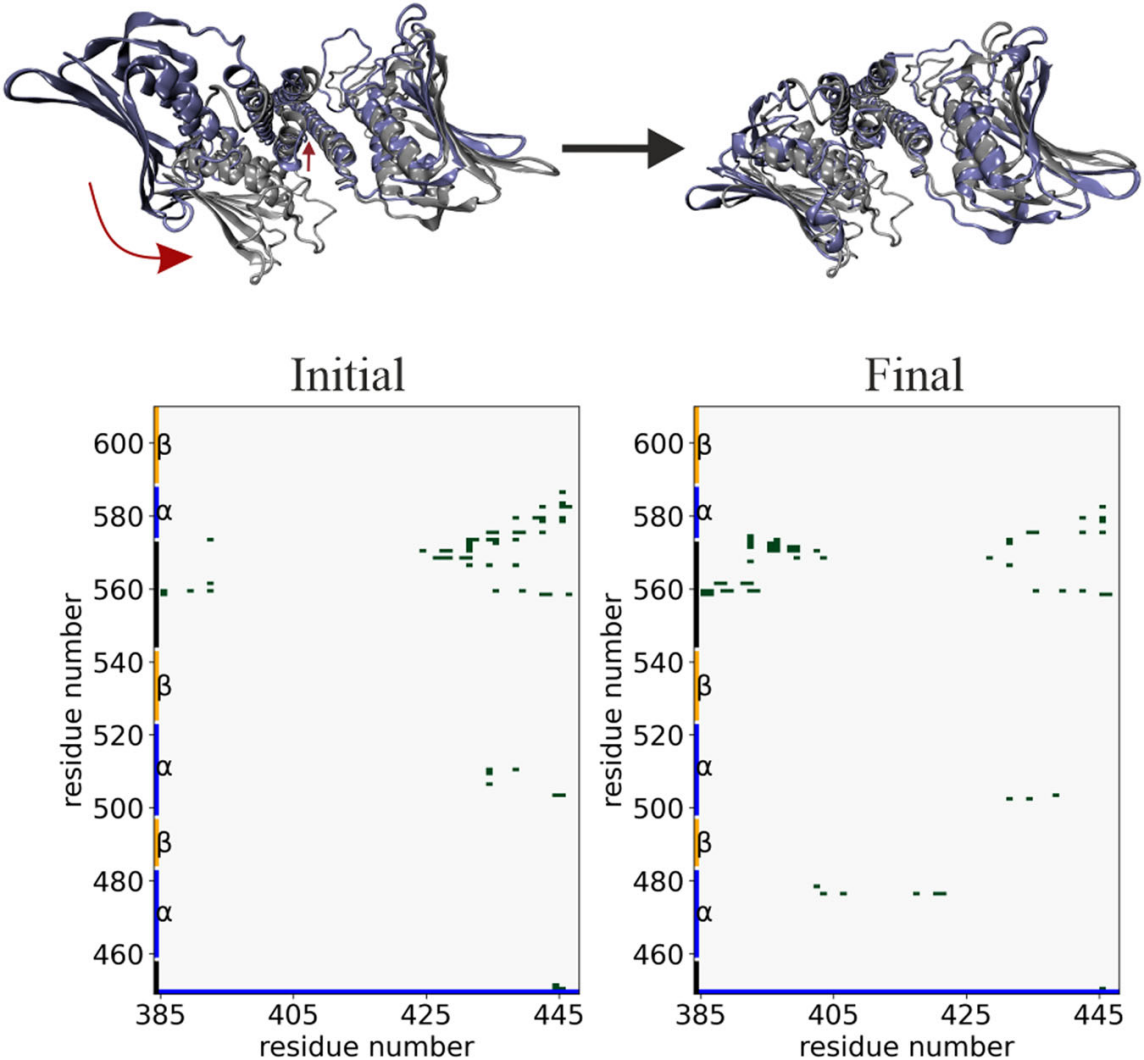
Step-wise activation pathway of histidine kinase. Top: The initial and final conformations (violet) from steered MD superimposed with the crystal active WalK histidine kinase (PDB ID 4U7O; grey). Bottom: Contact maps of CA–DHp interactions of the initial (left) and final (right) states. Each point on the contact map represents the presence (green square) or absence (no marking) of contacts between residues of the CA and DHp domains of chain B. Contacts are defined as non-bonded interactions between heavy atoms within a cut-off distance of 0.45 nm.

Significantly, we observed that the DHp helical bending lead to the rearrangement of the entire protein’s 3D structure such that the CA domain rotates concurrently. The CA domain rotates by 50 °, which is just 7 ° less than what had occurred when the RMSD bias was applied onto both domains. Additionally, the center of mass distance between the CA and DHp domains decreased from 2.55 nm to 1.95 nm. By the end of the simulation, the HK model had a backbone RMSD of 0.45 nm relative to WalK active state (PDB ID 4U7O) which indicates a large degree of structural similarity. Furthermore, important CA–DHp contacts including Arg568–Glu428, Arg434–Asp502 and Phe559–Asn388 were formed. Overall, there is a larger number of CA-DHp non-bonded interactions in the final state relative to the starting structure, see Fig. 5.

Thus we observed that the CA domain rotates in response to local force perturbations at the helical bundle of the DHp domain, which essentially mimics the forces exerted by ligand binding to N-terminal sensing domains in the full-length protein. These forces lead to a conformational transition of the DHp domain, which likely acts as a *switch* to trigger a transition from inactive to active state of the entire HK catalytic core in spite of the earlier observed large transition barrier.

### Phosphoryl transfer from ATP to His391

As soon as the active state is established, an autophosphorylation reaction takes place, consisting of a transfer of the *γ*-phosphate group from ATP to the N*ϵ* atom of His391 in the DHp domain, followed by a deprotonation of His391. This complex reaction is only possible with His391 in the HID protonation state, which was considered in the current simulations also: His391 can be phosphorylated on the unprotonated N*ϵ* atom, while the protonated N*δ* is stabilized by a strong hydrogen bridge to the Glu392 side chain. Another prerequisite for the autophosphorylation is the presence of an Mg^2+^ cation, which in this work was always found coordinating with six oxygen atoms after the QM/MM equilibration (one oxygen each in the side chain of Asn541, in the *γ*-phosphate, *β*-phosphate and *α*-phosphate of ATP as well as in two water molecules). Its mechanism cannot be studied by experimental means in any feasible way, while it poses a serious challenge for a computational investigation, requiring an approach that is both, sufficiently accurate and efficient.

**Fig. 6:**
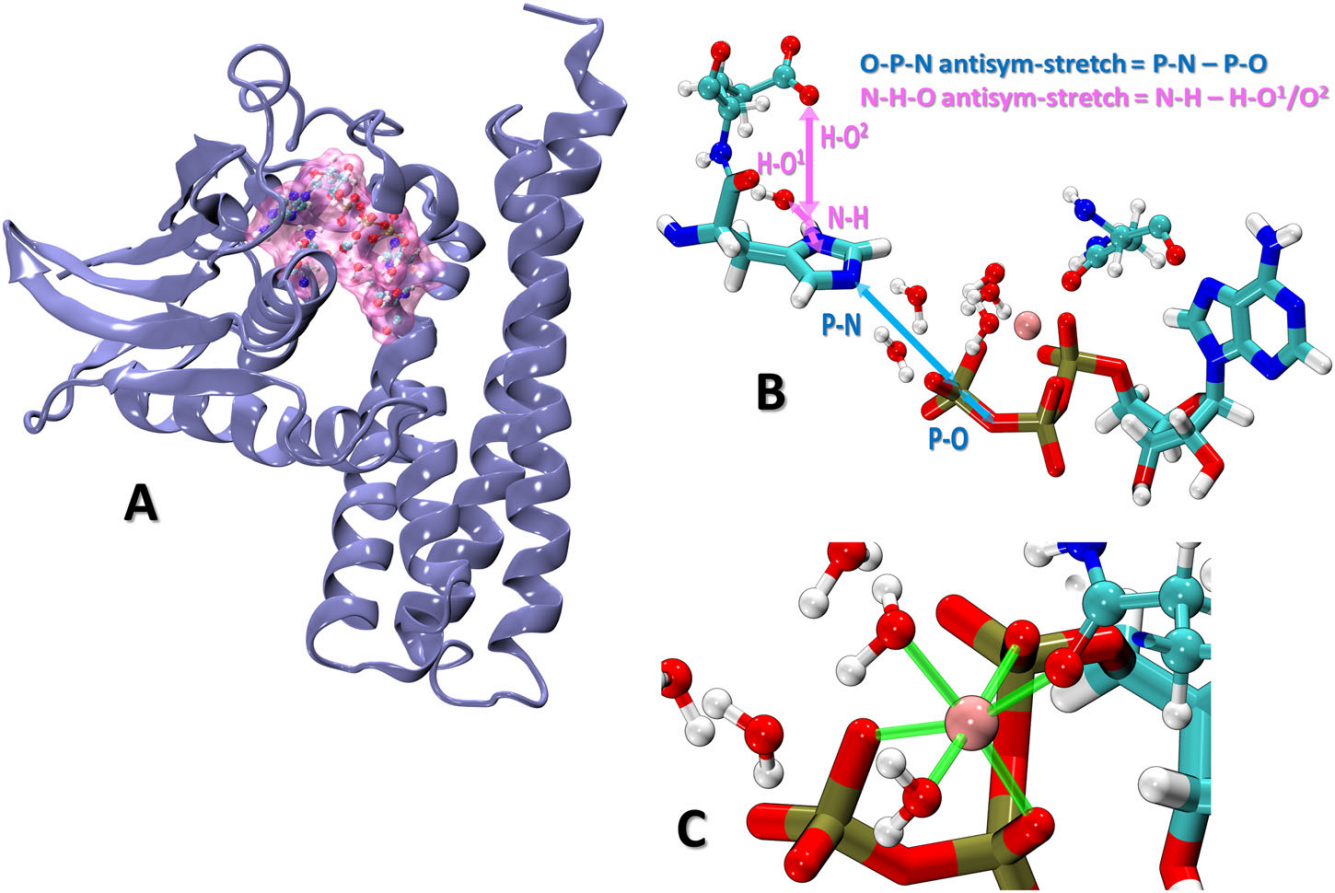
Model of WalK used as the initial structure for QM/MM metadynamics simulations of autophosphorylation. **A** The active structure adopted from PDB ID 4U7O with the non-reacting ATP-binding domain truncated; location of the reaction center highlighted in pink. **B** The QM region covering the reaction center; the antisymmetric stretch CVs presented in blue and pink. **C** Coordination sphere of the magnesium cation in the reactant structure involves three ATP oxygen atoms (one from each of the *α*-, *β*- and *γ*-phosphate groups, going from top-right to bottom-left), the oxygen of the side chain of Asn541, and two water oxygens.

The methodical choice taken in this work is a QM/MM multiple-walker metadynamics simulation employing the semi-empirical density-functional approach DFTB as the quantum chemical method. This easily parallelizable protocol makes it possible to reach microsecond sampling, while the accuracy approaches 1 kcal/mol due to a reparametrization of the P–N repulsive potential of DFTB. The QM region consisted of the side chains of His391 and Asn541, the ATP molecule with the coordinated Mg^2+^ ion, five nearby water molecules and a suitable proton acceptor, see Fig. 6 (a detailed discussion for the influence of the Mg^2+^ ion can be found in the SI). Two different simulations were performed, differing in the identity of the proton acceptor: the side chain of Glu392 in system 1, or an OH^-^ ion as proton acceptor in system 2. The metadynamics simulations involved two collective variables (CV) to describe the progress of the chemical reactions and express the potentials of the mean force (PMF): The O–P–N antisymmetric stretch [i.e., difference of the distances P*γ*(ATP)–O*β*(ATP) and P*γ*(ATP)–N*ϵ*(His391)] describes the transfer of the phosphoryl group, while the N–H–O antisymmetric stretch, [i.e., the difference of distances N*δ*(His391)–N*δ*(His391) and H*δ*(His391)–O(proton acceptor)] describes the transfer of the proton to the acceptor, see Fig. 6B. For illustration, a negative O–P–N means that the *γ*-phosphate group has transferred from ATP to His391, and a positive N–H–O denotes a completed proton transfer to the acceptor.

During the metadynamics simulation, the coordination sphere of Mg^2+^ always involved five or six ligands. In the final product, Mg^2+^ is coordinated with oxygens of three different water molecules, those of the *β*-phosphate and *α*-phosphate groups of ATP and, interestingly, an oxygen of the phosphohistidine. To further investigate the role of Mg^2+^ in the reaction, we additionally performed a restrained 2D QM/MM metadynamics simulation as follows: Five ligands of the coordination sphere of the Mg^2+^ were kept fixed (in accordance to product structure) and an additional water molecule (sixth ligand) was allowed to freely move in and out of the coordination sphere of Mg^2+^ and we monitored the free energy. The obtained PMF shown in Fig. S9 reveals a tiny energy difference between the coordination numbers of five and six, as well as a very low barrier of 2 kcal/mol to the un- and re-binding of the sixth ligand. This could mean that pHis is (almost) free to be released from the coordination sphere, and that would help the processes following the phosphoryl transfer: The protein domains could easily move away from each other if the coordination bond to be broken is not quite as strong.

### Mechanism of His Phosphorylation, and Nature of the Transition State

**Fig. 7:**
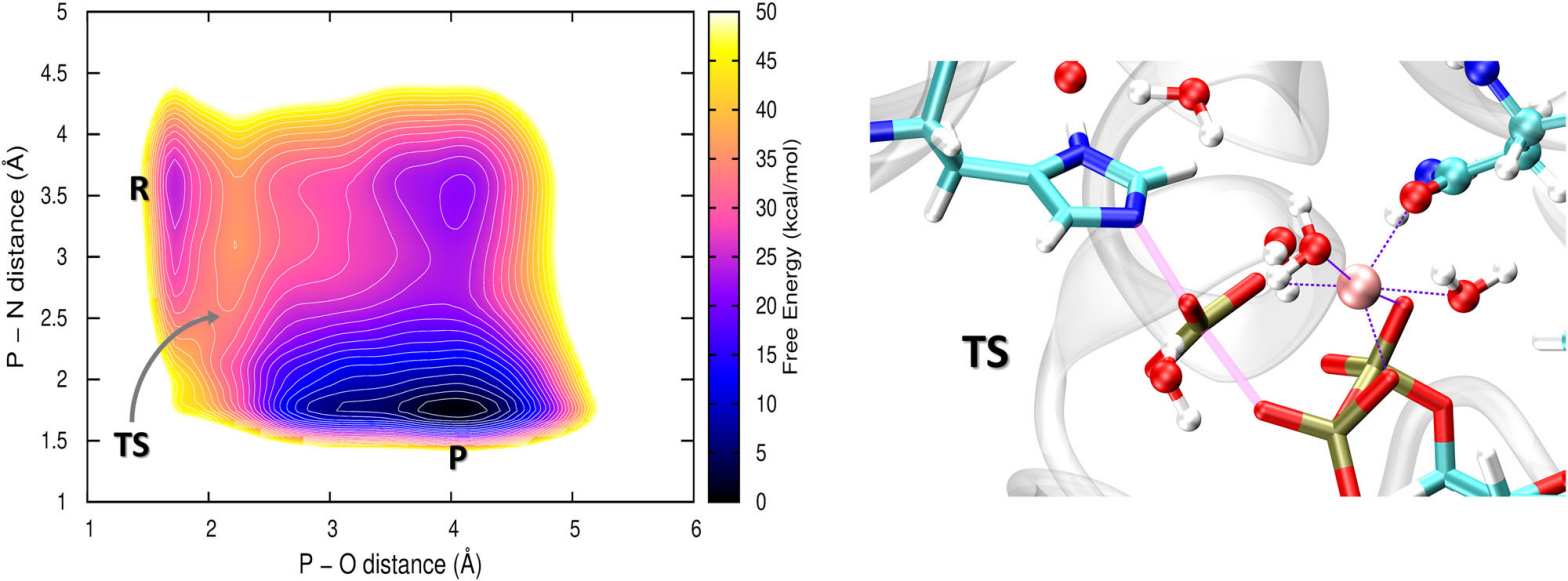
Results from 2D QM/MM metadynamics simulation using the distances P–N and P–O as CVs. Left: The 2D potentials of the mean force; free energy color coded in kcal/mol, and contour lined separated by 2 kcal/mol. Bottom: A representative transition state structure from that simulation; highlighted are P–N and P–O distances (thick solid lines) and the six coordination bonds to the Mg^2+^ ion (thin dashed lines).

To analyze the appearance of the transition state, another QM/MM metadynamics simulation was performed. This 2D metadynamics simulation used a pair of CVs designed to describe the phosphoryl transfer: the distances P*γ*(ATP)–N*ϵ*(His391) and P*γ*(ATP)–O*β*(ATP). The resulting PMF is shown in Fig. 7A. In the PMF can be observed both the associative (formation of a five-fold hyper-valent phosphate transition state, tight TS) and the dissociative (formation of metaphosphate, loose TS) mechanisms^56,57^. It appears, though, that the associative mechanism corresponds to the minimum energy pathway, with the tight TS yielding a (lower) energy barrier of 8 kcal/mol above the reactant. A very similar barrier height will be found in the simulation of the entire chemical step below, indicating that this is in fact the real mechanism.

The structure of the tight TS, which exhibits a five-fold hypervalent state of the phosphorus atom, as obtained from the simulation is shown in Fig. 7B. Interestingly, the bonding on the phosphorus atom is asymmetric, with the P–O distance of 2.17 Å being markedly shorter than the P–N distance of 2.53 Å as shown in Fig. 7B. This might be caused by the asymmetric composition of the coordination sphere of the Mg^2+^ ion, with the oxygens of the negatively charged phosphate on one side and the oxygens of the neutral Asn541 and waters on the other.

It is interesting to note that a certain asymmetry was found for the phosphoryl transfer reaction in the DesK:DesR HK:RR complex from *B. subtilis*^58^. Just like the autophosphorylation site of WalK, the phosphoryl transfer site of DesK:DesR contains an Mg^2+^ ion, and the authors argued that the location of Mg^2+^ closer to the RR-Asp being phosphorylated suppresses the backward phosphoryl transfer, making the reaction unidirectional. In the authophosphorylation of WalK, the Mg^2+^ ion is clearly located close to the ATP (later ADP) molecule all the time because it is by far the most attractive interaction partner, but there is a different sort of asymmetry in the TS: the P–O distance is much shorter than the P–N distance.

Trajtenberg et al. further argued that the distance between the phoshophoryl donor and acceptor modulates the thermodynamics of the reaction, via the different capability of the Mg^2+^ ion to stabilize either molecule or both^59^: At longer distance, Mg^2+^ can only stabilize either the phospho-reactant or the product, leading to (larger) free energy difference between them. At shorter distance, Mg^2+^ can interact with and stabilize both, equalizing the reaction free energy somewhat. In the current case of WalK, however, this relationship appears less important because the reaction appears down-the-hill (unlike the phosphoryl transfer from the HK to the RR investigated in Ref. ^59^) due to the energy gain from the deprotonation of phosphorylated His391.

### The chemical step is exergonic and base-dependent

**Fig. 8:**
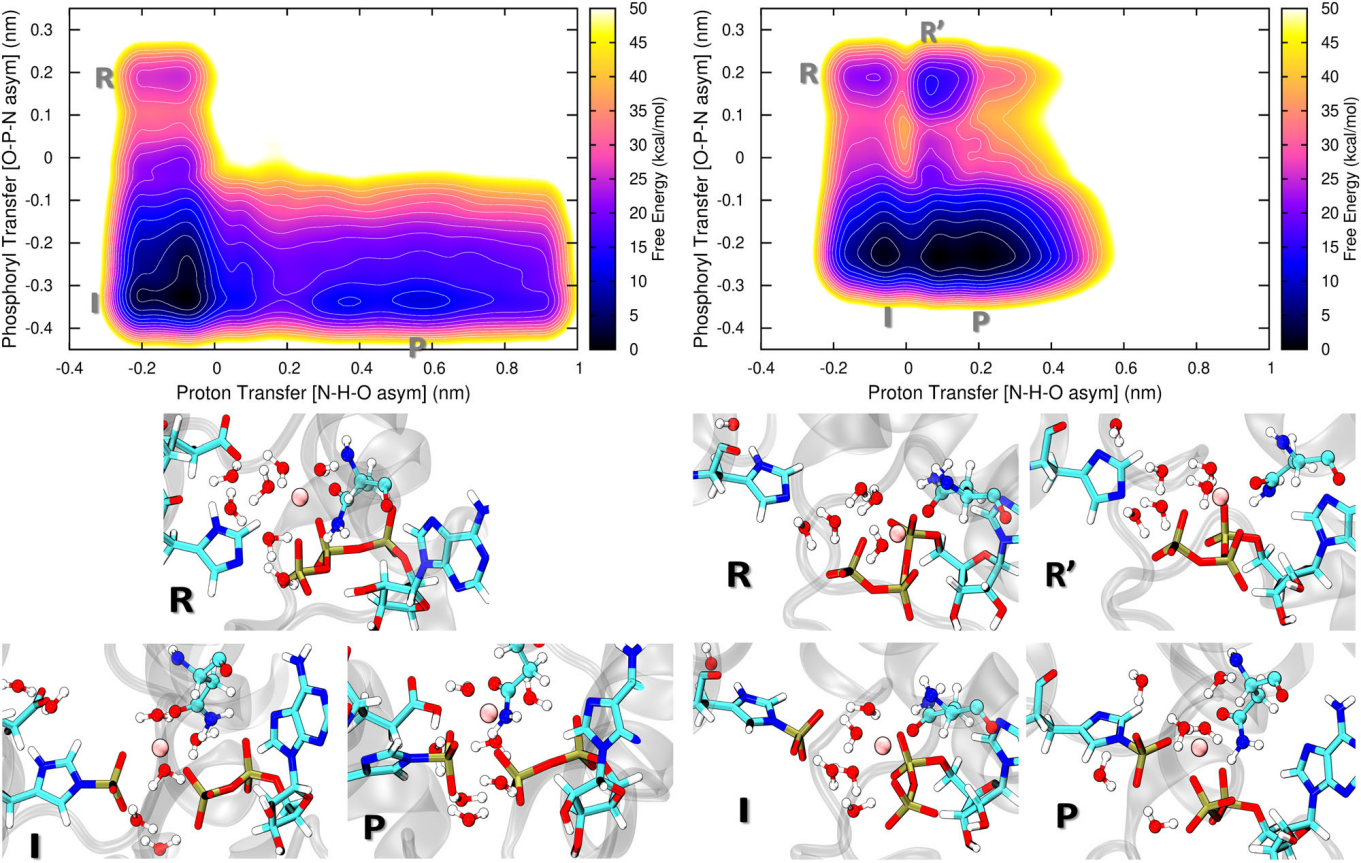
Results from the 2D QM/MM metadynamics simulations of autophosphorylation of WalK, using the antisymmetric stretches N–H–O and O–P–N as CVs. There are two different simulations: one involving the side chain of Glu392 as the proton acceptor, and the other with an OH^−^ ion playing that role. Top: Potentials of the mean force for the phosphorylation reaction. The free energy is color-coded, and the spacing of contour lines is 3 kcal/mol. Bottom: Representative structures of the states found on the PMF: R—reactant, R'—intermediate (His391 is deprotonated and unphosphorylated), I—intermediate (protonated phosphorylated His391), P—final product (deprotonated phosphohistidine).

We modelled the reaction considering the conserved Glu392, which is in a close contact with His391, as the proton acceptor. A good convergence of the resulting free energy surface (FES) shown in Fig. 8 (left) is indicated by the analysis presented in Fig. S10; no change in the resulting FES is visible after 700 ns of QM/MM simulation time. Passing over a barrier of 8 kcal/mol, the phosphoryl transfer leads to a protonated phosphohistidine intermediate, lying 20 kcal/mol below the reactant. Then, a nearly barrierless proton transfer from His391 to Glu392 leads to a final product that lies 13 kcal/mol above the protonated intermediate. This indicates that a stronger base is needed as the final proton acceptor to make the reaction sequence exergonic.

To this end, an OH^-^ ion was placed near the H*δ* atom of His391, which is the proton to be transferred. A new QM/MM metadynamics simulation was performed, see Fig. 8 (right) for the resulting free energy surface. Also, the analysis in Fig. S11 shows a good convergence of the FES after 700 ns of the QM/MM sampling. The final product lies less than 1 kcal/mol below the intermediate, and is a global minimum of free energy now. The energy barrier to the reaction sequence of 8 kcal/mol is identical to the previous case where considering a glutamate as the proton acceptor. The reason for this is that the higher barrier applies to the phosphoryl group transfer from ATP to histidine, which is exactly the same process in both cases. The subsequent proton transfer passes over a much lower barrier of 4 kcal/mol.

## Discussion

HK proteins and their role in TCS signal transduction cascades in bacteria have been widely studied for decades, and structural details of their activation mechanism have emerged more recently^27,28,33,60^. While the structural details of the endpoint conformations, i.e., phosphorylation inactive and phosphorylation active, are known, knowledge on the dynamics of the transition, the coupling between the domains and the relationship between the conformational changes and the subsequent phosphoryl transfer reaction is still limited. In a recent work, Olivieri et al. argued that the activation pathway likely occurs via a “walking” mechanism in which the CA gradually rotates relative to the DHp domain through the simultaneous breaking and formation of inter-domain contacts^12^. The alternative “release and rebind” pathway had a significantly higher energy barrier and hence was unfavorable. A study on CpxA investigated the influence of diphosphorylation of the individual monomers^61^ and suggested effects on the equilibrium constant for autophosphorylation vs. formation of ATP.

In the present study, we aimed to build upon past studies and explore the coupled motions of the DHp and CA domains following signal detection in WalK HK. We proposed two distinct activation pathways: (1) helical bending of the DHp domain and CA rotation is concerted or (2) occurs via a step-wise mechanism. Our work supports the hypothesis that the large-scale conformational changes of the subdomains are tightly coupled and therefore the activation of the kinase core is a concerted process.

From many enhanced sampling techniques^62^, we chose metadynamics as the endpoint crystal structures are available and we had an intuition of the transition pathway.

The free energy change during the concerted activation pathway determined from the metadynamics simulation is +10 kcal/mol, and the free energy barrier is 16 kcal/mol. These values likely represent upper bounds as ligand binding might bring about negative contributions as seen in the case of adenylate kinase^63^. The study by Olivieri et al. found quite similar energetics of the conformational transition^12^, in spite of the differences in their and our computational setups: theirs included a bound ATP molecule and used a different method (umbrella sampling) and CVs (the separate RMSDs with respect to the inactive and active conformations), indicating that the resulting energetics may be robust, independent of these computational details. Also, Trajtenberg et al. recently proposed a high-energy phosphotransferase state for another HK, and the energy landscape observed here for the WalK HK shows that such a state may be a common feature present in HK proteins^59^.

In spite of the increase in inter-domain contact pairs, the final active conformation had a larger free energy than the inactive state. Many of the amino acids in the contact pairs are hydrophilic due to the presence of a polar or charged side chain. These interactions are weaker than hydrophobic interactions due to their tendency to also interact with the water molecules surrounding the protein.

Using steered MD, we investigated the alternative step-wise activation mechanism. When the force was applied exclusively to the DHp, the helical bending of DHp induced the rotation of the CA domain by 50 °. Although the rotation angle of the CA domain was slightly less than that from the concerted steered MD, His391 in the DHp was brought closer to the ATP-binding pocket, ready for the phosphoryl-transfer reaction. In addition, essential CA-DHp contacts were formed (Fig. 5). Therefore, the large-scale motions of the DHp and CA domain necessary for activation clearly occurred simultaneously.

In contrast, Marsico et al. demonstrated that while HAMP-DHp activation in the CpxA HK induces CA domain reorientation, in their study only approximately one third of the full transition is achieved^64^. A key difference between CpxA and WalK HK lies in the directionality of the autophosphorylation reaction: WalK HK undergoes cis-phosphorylation whereas CpxA HK undergoes trans-phosphorylation. For HK proteins that phosphorylate in cis, each monomer phosphorylates itself. Conversely, trans-phosphorylation occurs when each monomer in the dimer phosphorylates the other subunit. It is believed that whether phosphorylation occurs in cis or trans is dependent on the handedness of the hairpin loop that connects the individual DHp helices^20^. Moreover, our WalK HK model did not include HAMP as the crystal structure was not readily available. Therefore, the implications of the presence of a linker domain such as HAMP cannot be determined. Even given these caveats, our results hint at a more general signal transduction mechanism of physiological relevance for HK proteins, with specifics of the HK changing its details.

Regarding the chemical step of histidine phosphorylation, we performed two different semi-empirical QM/MM metadynamics simulations of the process combining the phosphoryl transfer from ATP to His391 and the proton transfer from His391 either to the initially deprotonated Glu392 or to an OH^−^ anion placed near His391. The resulting free energy surfaces converged after simulations were extended to 1 *μ*s. We observe a free energy barrier to the chemical reaction of 8 kcal/mol, independent of the identity of the proton acceptor, leading to a stable intermediate represented by protonated phosphorylated His391.

Adding this value to the free energy of the active conformation of HK (+10 kcal/mol), which represents the reactant state of the phosphoryl transfer reaction, leads to the overall free energy barrier of 18 kcal/mol. The experimentally reported catalytic rate of 0.027 min^− 1^^29^ corresponds, using simple transition state theory expression for rate $=kT/h\cdot \exp \left[-{E}_{{{\rm{A}}}}/kT\right]$, to an activation energy (barrier) of 22 kcal/mol. Such an agreement can be considered favorable, given that any uncertainties of the individual simulations (of the conformational transition and of the chemical step) in fact add up, as well as given that an approximate rate expression is used with an experimental catalytic rate. Since we here aim largely at qualitative interpretations of the observed energy relationships, the discussions of the thermodynamic and kinetic parameters remain valid even if their uncertainties are considered equal to the above “deviation” of our computational barrier from the converted experimental value, which is 4 kcal/mol.

Notably, the barrier opposing the conformational transition obtained from our classical simulations, of 16 kcal/mol, represents the major part of the overall barrier. Therefore, the conformational transition is the rate-limiting step of the whole catalytic activity, as long as that proceeds from the inactive protein structure in equilibrium. Note that this corresponds to the situation in an in vitro experiment, whereas the genuine in vivo process is being triggered by an energy input from the sensory domain reacting to a stimulus, and this also effectively reduces the barrier.

**Fig. 9:**
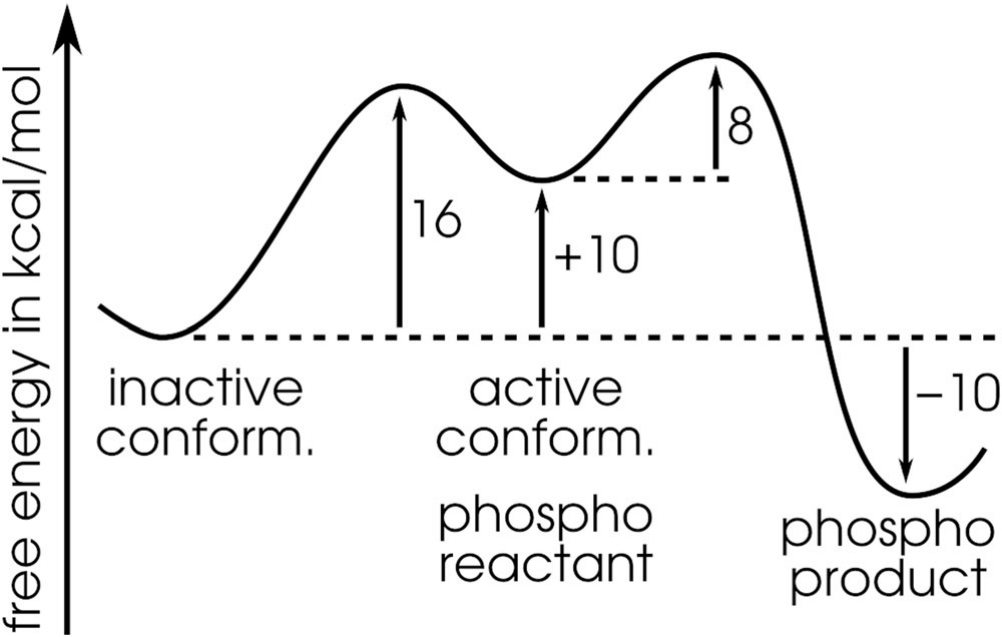
Overall free energy profile. The free energy profile of the entire autophosphorylation sequence including the conformational transition from the inactive to the active structure, and the phosphorylation of the His391 residue.

When terminating the reaction with the protonated Glu392, the product lies above the protonated pHis intermediate in free energy. This suggests that a stronger base has to be present as a real final proton acceptor, and this situation was considered in the second QM/MM metadynamics simulation, which had an OH^−^ anion located near His391. With this setup, the deprotonated phosphorylated His391 is a stable product, the species lying the lowest in free energy, 20 kcal/mol below the initial reactant, and even 10 kcal/mol below the inactive conformation of the protein, see Fig. 9. Therefore, for the phosphorylation to occur more readily, there has to be a strong base present as a final proton acceptor somewhere in the system. It is not necessary for it to be directly near the histidine, as long as a proton transfer pathway between the histidine and the proton acceptor is available. No matter what the pathway is like, the reaction energy will likely be similar; note that proton transfer may occur along rather long “water wires” exhibiting low energy barriers^65^.

We like to emphasize that the two simulations performed with different proton acceptors serve different purposes: The simulation with Glu392 as the acceptor shows the phosphorylation followed by proton transfer to Glu, which is likely the first step of the potentially complex deprotonation of the histidine. The other simulation with OH^−^ representing a general strong base aims to estimate the reaction energy of the entire process, involving a real final proton acceptor. We do not claim that an OH^-^ ion is genuinely present in close proximity to His391; most likely, it is not. The former simulation rules out the deprotonated unphosphorylated His391 (R’) as a viable intermediate, as it was suggested earlier to be an “activated” phosphoacceptor^28^, because it lies too high in energy in the realistic pathway proceeding via a protonated Glu392. The latter simulation in turn reveals that the chemical step of the autophosphorylation is in fact a down-the-hill process.

Clausen et al. showed that the rate of autophosphorylation in WalK increases with increasing pH, indicating the need for a strong base present as a proton acceptor^29^. In agreement with that, our simulation performed with and without an OH^-^ present provide a means to quantify the effect of a generic strong base available in the system, on the energetics and kinetics of the reaction. Prior computational studies of WalK and CpxA involved computationally considerably more costly DFT methods, which limited these studies to sub-nanosecond time scales^12,64^. The main finding on WalK was the “tight coupling” of the chemical step with the preceding conformational transition, whereby the protonation of His391 is prevented, which would otherwise hinder phosphorylation. The largely increased sampling in our current study unveils quantitative free energies surfaces, making it possible to explore the reaction mechanism in more detail. What we have learnt about the autophosphorylation, consisting of the phosphoryl-group transfer from ATP to His391 followed (at some point) by the deprotonation of pHis, leaves us with two possible scenarios of the whole process: One possibility is that pHis deprotonates immediately after the phosphoryl transfer has taken place. The first proton acceptor, Glu392 acts like a proton relay before the proton eventually is transfered to a sufficiently strong base (here represented by an OH^−^ anion), which need not be located directly next to pHis. In vivo, that strong base might be, for instance, a suitable titratable molecule present in the solution, or an area of local basic environment in the cytosol. The other conceivable scenario is that the base does not act in this step, and pHis does not deprotonate. Then, the next phosphorylation reaction, which is the phosphoryl transfer from pHis to the conserved Asp residue on the RR WalR, would have to proceed from the protonated pHis. Future work will need to answer whether this process would run spontaneously and if so, whether the energy barrier would be low enough to allow for reasonably favorable kinetics of the process.

Looking at the big picture of the phosphorylation cascade in TCS, the autophosphorylation of His391 will be followed by the transfer of the phosphoryl group from His391 to the conserved aspartate residue on the WalR RR. The final product of the reaction sequence in this study, the WalK protein in an active state containing a deprotonated phosphorylated His391, represents a quite stable minimum on the free energy surface, yet still phosphohistidine is a relatively unstable, high-energetic species. This means that the energy of ATP hydrolysis has not been fully released, and is still available to drive consecutive processes, of which the first is a phosphoryl group transfer to the conserved Asp in RR.

It appears likely that the dynamics and energetics of WalK autophosphorylation found here are valid not only for a single HK, rather, broadly representative of the HK autophosphorylation mechanism. We base this on the fact that direct coupling analysis of vast sequence alignments of HK proteins identified highly correlated residue pairings between DHp and CA domains, that later were identified to be in close proximity in individual structural examples of either inactive or active conformation of the HK protein^11^. Thus, the vast majority in sequence alignments included in this study have to have similar active and inactive conformations, in which these contacts can be realized. By extension, we also anticipate similar dynamics in the transition between active and inactive conformations.

## Conclusion

The initial processes in the signalling cascade of the WalK HK have been explored by means of a multi-scale simulation approach. Free and steered classical MD simulations were used to describe the conformational transition leading to the activated state. The interchanging patterns of favorable contacts between amino acid residues were analysed extensively, with emphasis on the contacts between the domains CA and DHp. Importantly, combination of two different simulation protocols made it possible to discover a correlation of the motions of the domains DHp and CA domains: Driving a conformation transition solely in the DHp domain induced a concurrent rotation of the CA domain also, and the process led to the experimentally known activated conformation. This thus appears to be one of a series of conformation coupling occurrences in a HK phosphorylation cascade.

The structure of the activated state of WalK HK served as a basis for an investigation of the autophosphorylation reaction, in which the *γ*-phosphate group of an ATP molecule bound to the CA domain is transferred to the His391 residue of the DHp domain. The applied semiempirical QM/MM MD multiple-walker metadynamics made it possible to achieve microsecond sampling and draw a reliable picture of the mechanism and energetics of the process. The reaction was shown to proceed via a penta-coordinated transition state to a protonated phosphohistidine intermediate, which is consequently deprotonated in favor of a suitable nearby base. The role of the basicity of the final proton acceptor was also described quantitatively.

The processes under study represent the first two steps of a long phosphorylation sequence in TCS, with the considered final product being still merely a high-energy intermediate in the sequence. The combined process is very slow, as known from a large body of previous experimental research, and in accordance with that, the obtained combined potential of the mean force indicated an energy barrier of 18 kcal/mol as seen in Fig. 9. The conformational transition to the active structure constitutes the rate-determining step that contributes most of the energy barrier, and is endergonic. The phosphorylation step, on the other hand, exhibits down-the-hill energetics, with the exact shape being dependent on the nature of the final proton acceptor. When considering OH^−^ in that role, the overall process combining the conformational transition and the phoshphoryl transfer reaction exhibits a reaction free energy of  −10 kcal/mol. That draws a picture with of an exergonic process accompanied by a high energy barrier, in agreement with and extending the current state of knowledge of the reaction.

## Supplementary information

Supplementary Information

## Data Availability

The authors declare that the data supporting the findings of this study are available within the paper and its Supplementary Information files. Should any raw data files be needed they are available from the corresponding author upon reasonable request.

## References

[1] Ulrich, L. E. & Zhulin, I. B. The MiST2 database: a comprehensive genomics resource on microbial signal transduction. *Nucleic Acids Res.* **38**, D401–D407 (2010). 19900966 10.1093/nar/gkp940 PMC2808908

[2] Krell, T. et al. Bacterial sensor kinases: diversity in the recognition of environmental signals. *Annu. Rev. Microbiol.* **64**, 539–559 (2010). 20825354 10.1146/annurev.micro.112408.134054

[3] Zschiedrich, C. P., Keidel, V. & Szurmant, H. Molecular mechanisms of two-component signal transduction. *J. Mol. Biol.* **428**, 3752–3775 (2016). 27519796 10.1016/j.jmb.2016.08.003 PMC5023499

[4] Igarashi, M. et al. Waldiomycin, a novel WalK-histidine kinase inhibitor from *Streptomyces sp*. MK844-mF10. *J. Antibiot.* **66**, 459–464 (2013). 10.1038/ja.2013.33 23632918

[5] Okada, A. et al. Walkmycin B targets WalK (YycG), a histidine kinase essential for bacterial cell growth. *J. Antibiot.* **63**, 89–94 (2010). 10.1038/ja.2009.128 20057515

[6] Weidenmaier, C., Goerke, C. & Wolz, C. *Staphylococcus aureus* determinants for nasal colonization. *Trends Microbiol.* **20**, 243–250 (2012). 22494802 10.1016/j.tim.2012.03.004

[7] Turner, N. A. et al. Methicillin-resistant *Staphylococcus aureus*: an overview of basic and clinical research. *Nat. Rev. Microbiol.* **17**, 203–218 (2019). 30737488 10.1038/s41579-018-0147-4 PMC6939889

[8] Raineri, E. J. M., Altulea, D. & van Dijl, J. M. Staphylococcal trafficking and infection—from ‘nose to gut’ and back. *FEMS Microbiol. Rev.* **46**, fuab041 (2021). 10.1093/femsre/fuab041 PMC8767451 34259843

[9] Bleul, L., Francois, P. & Wolz, C. Two-component systems of *S. aureus*: Signaling and sensing mechanisms. *Genes* **13**, 34 (2022). 10.3390/genes13010034 PMC8774646 35052374

[10] Schug, A., Weigt, M., Onuchic, J. N., Hwa, T. & Szurmant, H. High-resolution protein complexes from integrating genomic information with molecular simulation. *Proc. Natl. Acad. Sci. USA* **106**, 22124–22129 (2009). 20018738 10.1073/pnas.0912100106 PMC2799721

[11] Dago, A. E. et al. Structural basis of histidine kinase autophosphorylation deduced by integrating genomics, molecular dynamics, and mutagenesis. *Proc. Natl. Acad. Sci. USA* **109**, E1733–E1742 (2012). 22670053 10.1073/pnas.1201301109 PMC3387055

[12] Olivieri, F. A. et al. Conformational and reaction dynamic coupling in histidine kinases: Insights from hybrid QM/MM simulations. *J. Chem. Inf. Model.* **60**, 833–842 (2020). 31923359 10.1021/acs.jcim.9b00806

[13] Cheng, R. R., Morcos, F., Levine, H. & Onuchic, J. N. Toward rationally redesigning bacterial two-component signaling systems using coevolutionary information. *Proc. Natl. Acad. Sci. USA* **111**, E563–E571 (2014). 24449878 10.1073/pnas.1323734111 PMC3918776

[14] Jacob-Dubuisson, F., Mechaly, A., Betton, J.-M. & Antoine, R. Structural insights into the signalling mechanisms of two-component systems. *Nat. Rev. Microbiol.* **16**, 585–593 (2018). 30008469 10.1038/s41579-018-0055-7

[15] Takada, H. & Yoshikawa, H. Essentiality and function of WalK/WalR two-component system: the past, present, and future of research. *Biosci. Biotechnol. Biochem.* **82**, 741–751 (2018). 29514560 10.1080/09168451.2018.1444466

[16] Buschiazzo, A. & Trajtenberg, F. Two-component sensing and regulation: how do histidine kinases talk with response regulators at the molecular level? *Annu. Rev. Microbiol.* **73**, 507–528 (2019). 31226026 10.1146/annurev-micro-091018-054627

[17] Pirrung, M. C. Histidine kinases and two-component signal transduction systems. *Chem. Biol.* **6**, R167–R175 (1999). 10375545 10.1016/S1074-5521(99)80044-1

[18] Dikiy, I. et al. Insights into histidine kinase activation mechanisms from the monomeric blue light sensor el346. *Proc. Natl. Acad. Sci. USA* **116**, 4963–4972 (2019). 30808807 10.1073/pnas.1813586116 PMC6421462

[19] Bhate, M. P., Molnar, K. S., Goulian, M. & DeGrado, W. F. Signal transduction in histidine kinases: insights from new structures. *Structure* **23**, 981–994 (2015). 25982528 10.1016/j.str.2015.04.002 PMC4456306

[20] Ashenberg, O., Keating, A. E. & Laub, M. T. Helix bundle loops determine whether histidine kinases autophosphorylate in cis or in trans. *J. Mol. Biol.* **425**, 1198–1209 (2013). 23333741 10.1016/j.jmb.2013.01.011 PMC3636764

[21] Igo, M. M., Ninfa, A. J., Stock, J. B. & Silhavy, T. J. Phosphorylation and dephosphorylation of a bacterial transcriptional activator by a transmembrane receptor. *Genes Dev.* **3**, 1725–1734 (1989). 2558046 10.1101/gad.3.11.1725

[22] Wang, C. et al. Mechanistic insights revealed by the crystal structure of a histidine kinase with signal transducer and sensor domains. *PLoS Biol.* **11**, e1001493 (2013). 23468592 10.1371/journal.pbio.1001493 PMC3582566

[23] Fukushima, T. et al. A role for the essential YycG sensor histidine kinase in sensing cell division. *Mol. Microbiol.* **79**, 503–522 (2011). 21219466 10.1111/j.1365-2958.2010.07464.x PMC3556490

[24] Fabret, C. & Hoch, J. A. A two-component signal transduction system essential for growth of *Bacillus subtilis*: implications for anti-infective therapy. *J. Bacteriol.* **180**, 6375–6383 (1998). 9829949 10.1128/JB.180.23.6375-6383.1998 PMC107725

[25] Martin, P. K., Li, T., Sun, D., Biek, D. P. & Schmid, M. B. Role in cell permeability of an essential two-component system in *Staphylococcus aureus*. *J. Bacteriol.* **181**, 3666–3673 (1999). 10368139 10.1128/JB.181.12.3666-3673.1999 PMC93842

[26] Quezada, C. M. et al. Structural and chemical requirements for histidine phosphorylation by the chemotaxis kinase CheA. *J. Biol. Chem.* **280**, 30581–30585 (2005). 15994328 10.1074/jbc.M505316200

[27] Mechaly, A. E., Sassoon, N., Betton, J.-M. & Alzari, P. M. Segmental helical motions and dynamical asymmetry modulate histidine kinase autophosphorylation. *PLoS Biol.* **12**, e1001776 (2014). 24492262 10.1371/journal.pbio.1001776 PMC3904827

[28] Casino, P., Miguel-Romero, L. & Marina, A. Visualizing autophosphorylation in histidine kinases. *Nat. Commun.* **5**, 3258 (2014). 24500224 10.1038/ncomms4258

[29] Clausen, V. A. et al. Biochemical characterization of the first essential two-component signal transduction system from *Staphylococcus aureus* and *Streptococcus pneumoniae*. *J. Mol. Microbiol. Biotechnol.* **5**, 252–260 (2003). 12867749 10.1159/000071077

[30] Cui, Q. Perspective: Quantum mechanical methods in biochemistry and biophysics. *J. Chem. Phys.* **145**, 140901 (2016). 27782516 10.1063/1.4964410 PMC5065567

[31] Wolanin, P. M., Thomason, P. A. & Stock, J. B. Histidine protein kinases: key signal transducers outside the animal kingdom. *Genome Biol.* **3**, 3013.1 (2002). 10.1186/gb-2002-3-10-reviews3013 PMC244915 12372152

[32] Celikel, R., Veldore, V. H., Mathews, I., Devine, K. M. & Varughese, K. I. Atp forms a stable complex with the essential histidine kinase walk (yycg) domain. *Acta Crystallogr. Sect. D: Biol. Crystallogr.* **68**, 839–845 (2012). 22751669 10.1107/S090744491201373X PMC3388812

[33] Cai, Y. et al. Conformational dynamics of the essential sensor histidine kinase WalK. *Acta Crystallogr. D: Struct. Biol.* **73**, 793–803 (2017). 28994408 10.1107/S2059798317013043 PMC5633905

[34] Webb, B. & Sali, A. Comparative protein structure modeling using modeller. *Curr. Protoc. Bioinformatics* **54**, 5–6 (2016). 10.1002/cpbi.3 PMC5031415 27322406

[35] Hess, B., Kutzner, C., van der Spoel, D. & Lindahl, E. GROMACS 4: Algorithms for highly efficient, load-balanced, and scalable molecular simulation. *J. Chem. Theory Comput.* **4**, 435–447 (2008). 26620784 10.1021/ct700301q

[36] Abraham, M. J. et al. GROMACS: High performance molecular simulations through multi-level parallelism from laptops to supercomputers. *SoftwareX* **1–2**, 19–25 (2015). 10.1016/j.softx.2015.06.001

[37] Lindahl, E., Abraham, M., Hess, B. & van der Spoel, D. Gromacs 2020 manual. 10.5281/zenodo.3562512 (2020).

[38] Bonomi, M., Bussi, G., Camilloni, C., Tribello, G. A. & The PLUMED consortium. Promoting transparency and reproducibility in enhanced molecular simulations. *Nat. Methods* **16**, 670–673 (2019). 31363226 10.1038/s41592-019-0506-8

[39] Lindorff-Larsen, K. et al. Improved side-chain torsion potentials for the Amber ff99SB protein force field. *Proteins: Struct. Funct. Bioinf.* **78**, 1950–1958 (2010). 10.1002/prot.22711 PMC2970904 20408171

[40] Jorgensen, W. L., Chandrasekhar, J., Madura, J. D., Impey, R. W. & Klein, M. L. Comparison of simple potential functions for simulating liquid water. *J. Chem. Phys.* **79**, 926–935 (1983). 10.1063/1.445869

[41] Darden, T., York, D. & Pedersen, L. Particle–mesh Ewald: An method for Ewald sums in large systems. *J. Chem. Phys.* **98**, 10089–10092 (1993). 10.1063/1.464397

[42] Hess, B., Bekker, H., Berendsen, H. J. C. & Fraaije, J. G. E. M. LINCS: a linear constraint solver for molecular simulations. *J. Comput. Chem.* **18**, 1463–1472 (1997). 10.1002/(SICI)1096-987X(199709)18:12<1463::AID-JCC4>3.0.CO;2-H

[43] Bussi, G., Donadio, D. & Parrinello, M. Canonical sampling through velocity rescaling. *J. Chem. Phys.* **126**, 014101 (2007). 17212484 10.1063/1.2408420

[44] Parrinello, M. & Rahman, A. Polymorphic transitions in single crystals: A new molecular dynamics method. *J. Appl. Phys.* **52**, 7182–7190 (1981). 10.1063/1.328693

[45] Kubař, T., Welke, K. & Groenhof, G. New QM/MM implementation of the DFTB3 method in the Gromacs package. *J. Comput. Chem.* **36**, 1978–1989 (2015). 26238364 10.1002/jcc.24029

[46] Kubař, T. Gromacs – QM/MM interface for DFTB+. https://github.com/tomaskubar/gromacs-dftbplus (2022). Last accessed 18 March 2022.

[47] Kubař, T. DFTB+ – modified QM/MM interface. https://github.com/tomaskubar/dftbplus (2022). Last accessed 18 March 2022.

[48] Hourahine, B. et al. DFTB+, a software package for efficient approximate density functional theory based atomistic simulations. *J. Chem. Phys.* **152**, 124101 (2020). 32241125 10.1063/1.5143190

[49] Tribello, G. A., Bonomi, M., Branduardi, D., Camilloni, C. & Bussi, G. Plumed 2: New feathers for an old bird. *Comput. Phys. Commun.* **185**, 604–613 (2014). 10.1016/j.cpc.2013.09.018

[50] Gaus, M., Cui, Q. & Elstner, M. DFTB3: extension of the self-consistent-charge density-functional tight-binding method (SCC-DFTB). *J. Chem. Theory Comput.* **7**, 931–948 (2011). 10.1021/ct100684s PMC3509502 23204947

[51] Gaus, M., Goez, A. & Elstner, M. Parametrization and benchmark of DFTB3 for organic molecules. *J. Chem. Theory Comput.* **9**, 338–354 (2013). 26589037 10.1021/ct300849w

[52] Kansari, M., Eichinger, L. & Kubař, T. Extended-sampling QM/MM simulation of biochemical reactions involving P–N bonds. *Phys. Chem. Chem. Phys.* **25**, 9824–9836 (2023). 36975159 10.1039/D2CP05890A

[53] Gaus, M., Lu, X., Elstner, M. & Cui, Q. Parameterization of DFTB3/3OB for sulfur and phosphorus for chemical and biological applications. *J. Chem. Theory Comput.* **10**, 1518–1537 (2014). 24803865 10.1021/ct401002w PMC3985940

[54] Raiteri, P., Laio, A., Gervasio, F. L., Micheletti, C. & Parrinello, M. Efficient reconstruction of complex free energy landscapes by multiple walkers metadynamics. *J. Phys. Chem. B* **110**, 3533–3539 (2006). 16494409 10.1021/jp054359r

[55] Barducci, A., Bussi, G. & Parrinello, M. Well-tempered metadynamics: a smoothly converging and tunable free-energy method. *Phys. Rev. Lett.* **100**, 020603 (2008). 18232845 10.1103/PhysRevLett.100.020603

[56] Rosta, E., Kamerlin, S. C. & Warshel, A. On the interpretation of the observed linear free energy relationship in phosphate hydrolysis: a thorough computational study of phosphate diester hydrolysis in solution. *Biochemistry* **47**, 3725–3735 (2008). 18307312 10.1021/bi702106m

[57] Klähn, M., Rosta, E. & Warshel, A. On the mechanism of hydrolysis of phosphate monoesters dianions in solutions and proteins. *J. Am. Chem. Soc.* **128**, 15310–15323 (2006). 17117884 10.1021/ja065470t

[58] Trajtenberg, F. et al. Regulation of signaling directionality revealed by 3D snapshots of a kinase:regulator complex in action. *eLife* **5**, e21422 (2016). 27938660 10.7554/eLife.21422 PMC5231405

[59] Lima, S. et al. An allosteric switch ensures efficient unidirectional information transmission by the histidine kinase DesK from *Bacillus subtilis*. *Sci. Signal.* **16**, eabo7588 (2023). 36693130 10.1126/scisignal.abo7588

[60] Gushchin, I. et al. Sensor histidine kinase NarQ activates via helical rotation, diagonal scissoring, and eventually piston-like shifts. *Int. J. Mol. Sci.* **21**, 3110 (2020). 32354084 10.3390/ijms21093110 PMC7247690

[61] Bouillet, S., Wu, T., Chen, S., Stock, A. M. & Gao, R. Structural asymmetry does not indicate hemiphosphorylation in the bacterial histidine kinase cpxa. *J. Biol. Chem.* **295**, 8106–8117 (2020). 32094228 10.1074/jbc.RA120.012757 PMC7278341

[62] Wingbermühle, S. & Schäfer, L. V. Capturing the flexibility of a protein–ligand complex: Binding free energies from different enhanced sampling techniques. *J. Chem. Theory Comput.* **16**, 4615–4630 (2020). 32497432 10.1021/acs.jctc.9b01150

[63] Arora, K. & Brooks III, C. L. Large-scale allosteric conformational transitions of adenylate kinase appear to involve a population-shift mechanism. *Proc. Natl. Acad. Sci. USA* **104**, 18496–18501 (2007). 18000050 10.1073/pnas.0706443104 PMC2141805

[64] Marsico, F. et al. Multiscale approach to the activation and phosphotransfer mechanism of CpxA histidine kinase reveals a tight coupling between conformational and chemical steps. *Biochem. Biophys. Res. Commun.* **498**, 305–312 (2018). 28911864 10.1016/j.bbrc.2017.09.039

[65] Riccardi, D. et al. "Proton holes” in long-range proton transfer reactions in solution and enzymes: a theoretical analysis. *J. Am. Chem. Soc.* **128**, 16302–16311 (2006). 17165785 10.1021/ja065451j PMC2561195

[66] Jülich Supercomputing Centre. JUWELS cluster and booster: Exascale pathfinder with modular supercomputing architecture at Juelich Supercomputing Centre. *J. Large-scale Res. Facilities* **7**, A138 (2021).

