# Supplementary material for: Mechanism of activation and autophosphorylation of a histidine kinase
Source: Commun Chem. 2024 Sep 3;7:196. doi: 10.1038/s42004-024-01272-6 (PMC11371814; doi:10.1038/s42004-024-01272-6)
Supplement: Supplementary file 1 — Supplementary Information [file 42004_2024_1272_MOESM1_ESM.pdf]

Supplementary Information to Mechanism of activation and autophosphorylation of a histidine kinase

Mayukh Kansari[†1], Fathia Idiris[†2], Hendrik Szurmant[3], Tomáš Kubař[1] and Alexander Schug[4,5]

[1]Institute of Physical Chemistry, Karlsruhe Institute of Technology, Karlsruhe, Germany.
[2]Steinbuch Centre for Computing, Karlsruhe Institute of Technology, Karlsruhe, Germany.
[3]College of Osteopathic Medicine of the Pacific, Western University of Health Sciences, Pomona, California, USA.
[4]Jülich Supercomputing Centre, Forschungszentrum Jülich, Jülich, Germany.
[5]Faculty of Biology, University of Duisburg/Essen, Essen, Germany.
[†]contributed equally.

Contributing authors: kansarimayukh@gmail.com; fathia.idiris@kit.edu; hszurmant@westernu.edu; tomas.kubar@kit.edu; al.schug@fz-juelich.de;

This PDF file includes:

- Supplementary text
- Figs. S1 to S12
- SI References

Supplementary Methods – Molecular Dynamics Simulations

All MD simulations of WalK HK were conducted at 300 K using GROMACS 2020.4[1–3] patched with PLUMED v2.7[4], with AMBER99SB-ILDN force-field[5], and with a time step of 2 fs. Each HK state was solvated with the TIP3P water model[6]. Electrostatic interactions were calculated using the particle mesh Ewald (PME) method[7], with a direct cutoff of 1.0 nm and a grid spacing of 0.16 nm. A cutoff of 1.0 nm was used for the van der Waals interactions. All bonds involving hydrogen were constrained by the LINCS algorithm[8]. Each HK-water system was first minimized using the steepest decent algorithm for 1000 steps. Then, NVT equilibration was performed for 1 ns using the velocity-rescaling thermostat[9], allowing the system to reach temperature convergence. Once the temperature was stabilized, NPT equilibration was carried out for 1 ns with the Parrinello–Rahman barostat[10], effectively achieving pressure convergence.

Metadynamics

We ran a two-dimensional multiple walker metadynamics of the conformational transition using 12 walkers for an accumulated sampling of 1.7 μs. The initial structures were the previously equilibrated active and inactive structures, considere for six of the walkers each. The collective variables (CVs) considered here were ΔRMSD and COMTOR, which is the torsional angle defined by the centers of mass (COM) of four different parts of the protein (COM1: chain A residues 68–226, COM2: chain A residues 3–67, COM3: chain B residues 1–61, COM4: chain B residues 62–226). The metadynamics was run with a constant Gaussian bias height of 1.0 kJ/mol, and widths of 0.02 nm and 1.15°units in ΔRMSD and COMTOR, respectively.

The biases were deposited in every 1000th step, and were communicated between the individual walkers every 1500 steps. For presentation, the resulting PMF was projected to the ΔRMSD dimension (i.e., COMTOR was integrated out). The metadynamics simulation involved additional restraints: (i) The sum of both RMSDs (with respect to the inactive and the active structures, respectively – i.e., RMSD(inactive) + RMSD(active)) were restrained to values lower than 1.6 nm ('upper wall' of PLUMED) with a force constant of 1,500,000 $\mathrm{kJ\,mol^{-1}\,nm^{-2}}$. (ii) COMTOR was restrained to the interval between $-57.3°$ and $-171.8°$ ('lower' and 'upper walls') with a force constant of 15,000 $\mathrm{kJ\,mol^{-1}\,rad^{-2}}$.

Structural characterization – clustering and contact frequency maps

The important conformational states as the minima of the free energy. Then, five different representative structures were selected manually from the trajectory from the metadynamics simulation, corresponding to the states I, B, C, D and A (as given in Fig. 3 in the main text). Additional independent, unrestrained MD simulations were run starting from these structures. The trajectories from these simulations were analyzed in two ways: (i) Cluster analysis was performed on each trajectory, and the centroid of the cluster with the largest volume was considered as a representative structure of the state, and visualized. (ii) Contact frequencies between amino acid residues were obtained from all of the unrestrained simulations, and differential contact energy maps were constructed for the pairs of states B–C, C–D and D–A.

DHp-only Steered MD

Using the equilibrated inactive WalK histidine kinase in explicit water system as the initial structure, a time-dependent harmonic bias was applied to the Cα atoms of the DHp domain of both protomers.

$$V_{\mathrm{DHp}} = \frac{k}{2}\left(\mathrm{RMSD}(t) - \mathrm{RMSD}^*\right)^2. \tag{1}$$

Here, k is the spring constant, $\mathrm{RMSD}(t)$ is the instantaneous RMSD between the current coordinates and the reference structure. RMSD^* is a value that evolves linearly from the initial RMSD at the first steered MD step to the final target RMSD. At each time step, the structure was first aligned with the Cα atoms of the DHp domain and the β-regions of the active CA domain. Then, the RMSD was calculated for only the Cα atoms of the DHp domain. This steered MD simulation was run for 2 ns with a spring constant of 5000 $\mathrm{kJ\,mol^{-1}\,nm^{-2}}$. Preliminary test simulations with various force constants were trialled before determining that 5000 $\mathrm{kJ\,mol^{-1}\,nm^{-2}}$ was sufficient in driving the transition.

Position of the Magnesium Cation

The action of kinases generally requires the presence of a magnesium cation as a cofactor[11,12]. Since the crystal structure used here as the initial structure included a non-hydrolyzable ATP analog and no magnesium, it was necessary to proceed with care and find the right position of Mg^{2+}. In order to do so, the PDB was searched for both active and inactive structures of wild-type and mutant kinases that do have a coordinated Mg^{2+} cation. The cation was always found in a very similar position in the ATP binding domain in the structures of different HK proteins, assuming a coordination to an oxygen atom of the γ-phosphate group of the bound ATP. Therefore, to complete the preparation of the initial structure for QM/MM simulations, several structural

models were created, featuring an Mg^{2+} cation in slightly different positions close to the γ-phosphate of ATP. Importantly, during an equilibration period of QM/MM simulations, the Mg^{2+} cation was always found coordinating with the same six oxygen atoms (one each in the side chain of Asn541, in the γ-phosphate, β-phosphate and α-phosphate of ATP as well as in two water molecules). That eventually provided a suitable initial structure to start the metadynamics simulation.

Both the reactant state and the final product of the reaction feature the Mg^{2+} ion with a stable coordination sphere containing six ligands. In the course of the metadynamics simulation, the coordination sphere of Mg^{2+} oscillates between five and six ligands. Water molecules were found to engage in strong hydrogen bonding with the β-phosphate group of ATP and the phosphohistidine, and that is why they showed the propensity to at times decordinate from Mg^{2+}. We further investigated the coordination sphere of Mg^{2+} using an additional QM/MM metadynamics simulation, which included additional water molecules in the QM region to ensure that any nearby water molecule is able to fill up the vacancy in the coordination sphere. The resulting PMF in Fig. S2 shows a negligible energy difference between the coordination numbers of five and six, as well as a very low barrier of 2 kcal/mol to the un- and re-binding of the sixth ligand (a water molecule).

Supplementary Methods – QM/MM simulations

Preparation and MM Equilibration

We started from crystal structure PDB ID 4U7O[13], which is activated WalK histidine kinase. The structure contains a non-hydrolysable ATP analogue AN2 and no magnesium present. We modelled non-terminal missing loops, and modified AN2 to ATP using UCSF Chimera[14] interfaced with MODELLER[15]. An

Mg^{2+} ion was placed carefully in between the γ- and β-phosphate groups. After that, the other ATP-binding domain which, located far away from the DHp domain, was truncated to reduce the size of the system. Finally, the biomolecular complex was enclosed in a periodic box sized ca. $8 \times 8 \times 8$ nm^3, which was filled with water and electro-neutralized by the addition of nine sodium counterions. The density of the system was 1014 kg m^{-3}.

The AMBER99SB-ILDN force field was used to describe the protein[5], while the parametrization of ATP from Ref. 16 was employed. The solvent was represented with the TIP3P water model[17] and Åqvist's parameters for the counterions[18]. The electrostatic interactions were treated with PME[7,19], where the short-range contribution was cut-off at 1 nm. The Lennard-Jones interactions were cut-off at 1 nm. All of the QM/MM MD simulations used the leap-frog integrator[20] with a time step of 1 fs, while all bonds involving hydrogen atoms were constrained with LINCS[8].

First, the system in the entirely MM representation was energy minimized with steepest descents. Then, it was equilibrated for 10 ns maintaining the temperature of 300 K by means of the Bussi thermostat[21].

QM/MM Preparation

Two different QM/MM setups were prepared Using the final structure from the MM equilibration. The QM region was introduced, consisting of the reaction center and its nearest neighbourhood:

- **System 1 – Glu392 considered as the final proton acceptor:** The QM region contains the side chains of His391, Glu392 and Asn541, the ATP molecule, the Mg^{2+} ion and 5 water molecules (70 atoms in total).
- **System 2 – a hydroxyl ion considered as the final proton acceptor:** The QM region contains the side chains of His391 and Asn541, the

ATP molecule, Mg^{2+} ion, 5 water molecules, and an OH^- ion created by removing a proton from a water molecule (56 atoms in total).

The QM region was treated with the semi-empirical density-functional method DFTB3[22] employing the 3OB parameter set[23] augmented with a special parametrizations for the pair interactions P–O and P–N[24]. The QM–MM interactions were treated by means of electronic embedding, which involved our PME implementation[25]. The MM region was described with the same force fields as employed in the preceding MM equilibration, as specified above. All of the MD simulation parameters were kept also, and the prepared QM/MM system were equilibrated at 300 K for 1 ns. The QM/MM simulations were performed using a local version of GROMACS[1,2,26] interfaced with PLUMED[27] and a local version of DFTB+[28,29].

QM/MM Free Energy Calculations

Potentials of the mean force were generated by means of multiple walker[30] two-dimensional metadynamics[31] employing 96 individual simulations (walkers). An initial phase of 47 ns was run with a constant Gaussian height. The second phase involved a well-tempered metadynamics protocol[32].

Collective Variables

Two collective variables were employed in the metadynamics simulations as follows, see also Fig. 6 in the main text.

- **Phosphoryl transfer:** O–P–N antisymmetric stretch, which is the difference of the distances: P(γ-phosphate of ATP)–Nε(His391) − P(γ-phosphate of ATP)–O(β-phosphate of ATP)
- **Proton Transfer:** N–H–O antisymmetric stretch, which is the difference of the distances Nδ(His391)–Hδ(His391) − Hδ(His391)–O(OH^-/Glu392)

Metadynamics

In the normal metadynamics phase, the height of the biasing Gaussians deposited was 1.2 kJ mol^{-1}, and their width was 0.02 nm in both dimensions. In the consective well-tempered metadynamics phase, a bias factor of 80 and 70 was considered in the simulations conseridering Glu392 and a hydroxyl ion, respectively, as the final proton acceptor. In all cases, the period of bias deposition was 500 steps, and the biases were communicated between the individual walkers every 1000 steps.

Restraints

The following additional harmonic restrains were applied in the QM/MM simulation:

- All O–H bonds of QM water molecules were restrained to 0.1 nm length with a force constant of 15,000 kJ mol^{-1} nm^{-2}.
- The angle Nδ(His391)–P(γ-phosphate of ATP)–O(β-phosphate of ATP) was restrained to values higher than 172° with a force constant of 1500 kJ mol^{-1} rad^{-2} ('lower wall' of PLUMED).
- The distance P(γ-phosphate of ATP)–Nε(His391) was restrained to values lower than 0.35 nm ('upper wall' of PLUMED) with a force constant of 15,000 kJ mol^{-1} nm^{-2}.
- The distance P(γ-phosphate of ATP)–O(β-phosphate of ATP) was restrained to values lower than 0.40 nm ('upper wall') with a force constant of 15,000 kJ mol^{-1} nm^{-2}.
- In the simulation of System 1: the proton transfer CV (N–H–O antisymmetric stretch) was restrained to the interval between -0.2 and 0.7 nm ('lower' and 'upper walls') with a force constant of 1500 kJ mol^{-1} nm^{-2}.

- In the simulation of System 2: the proton transfer CV (N–H–O antisymmetric stretch) was restrained to the interval between −0.2 and 0.2 nm ('lower' and 'upper walls') with a force constant of 1500 kJ mol^{-1} nm^{-2}.

Supplementary Results – Inactive to Active State Conformational Transition

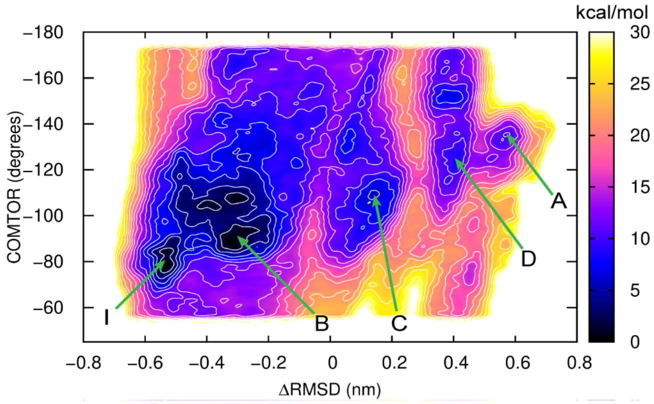

Figure S1 The original, two-dimensional PMF obtained from the 2D multiple walker metadynamics simulation of the conformational transition from the inactive to the active structure. The arrows pointing to the structural states are labeled in agreement with the labeling of the states introduced in Fig. 3 in the main text.

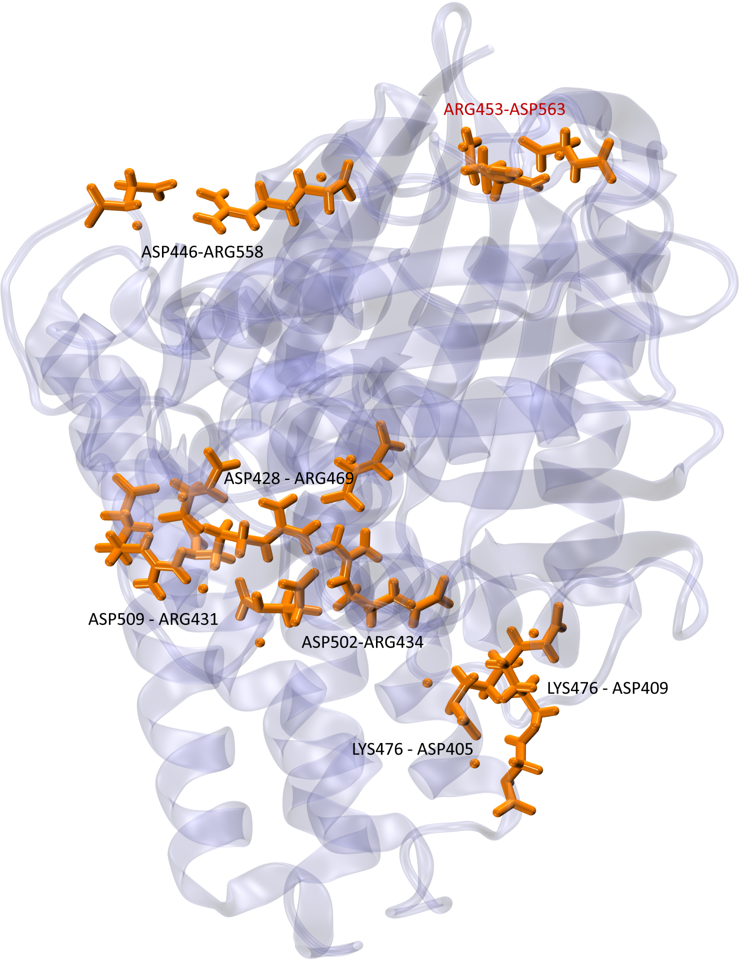

Figure S2 This is the side view of the active conformer, all seven salt-bridge interactions are shown here, residues participating in the interactions are highlighted in orange colour, text highlighted in red indicates inter-domain (CA-CA) salt-bridge interaction

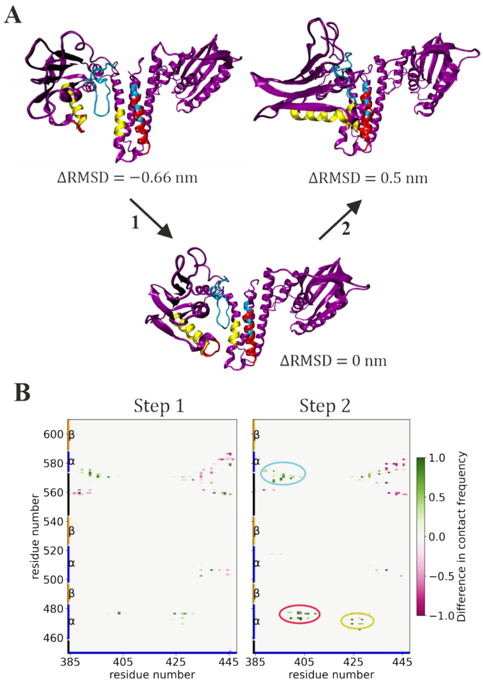

Figure S3 Concerted activation pathway of histidine kinase as observed in pilot umbrella sampling simulations. (A) Three structures from the transition pathway. Step 1 is the transition from the inactive state (ΔRMSD $= -0.66$ nm) to an intermediate (ΔRMSD $= 0$ nm) and step 2 is from this intermediate to the active state (ΔRMSD $= 0.5$ nm). Regions colored in red, yellow and blue are the areas where inter-domain contact pair interactions were favored in the active state. (B) Difference in contact frequency maps for steps 1 and 2. Three regions where contact pair formation was highly favorable are circled in red, yellow and blue as seen in A. Contacts are defined as non-bonded interactions between heavy atoms within a cut-off distance of 0.45 nm.

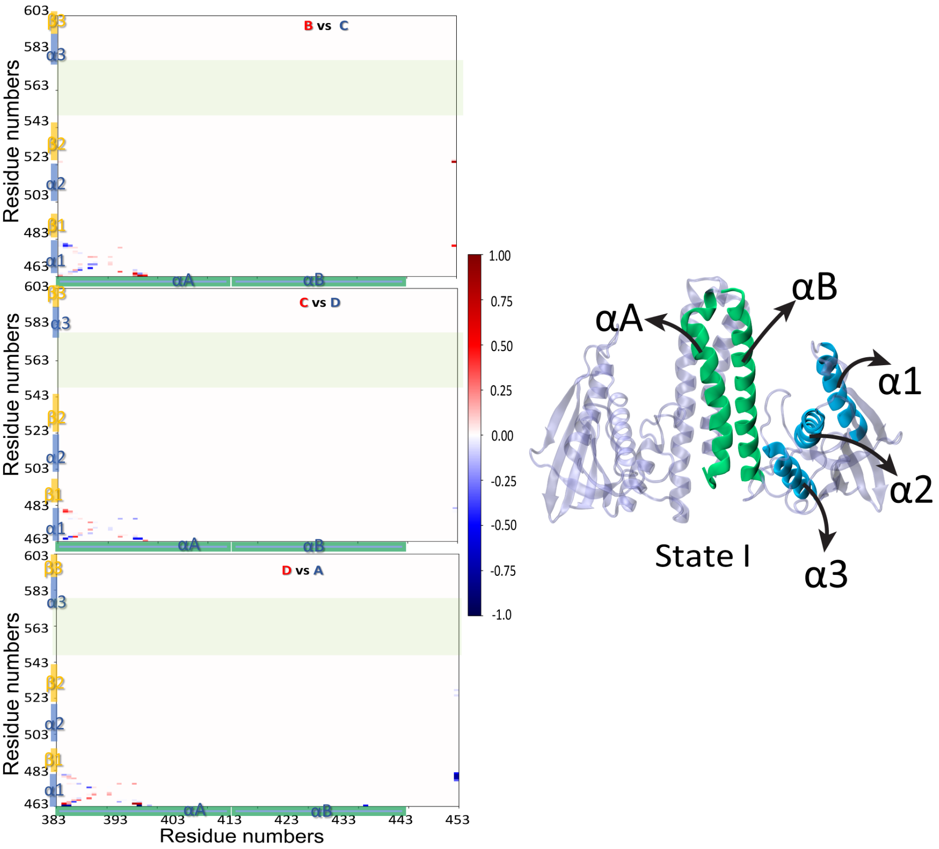

Figure S4 Differential map of frequencies of non-polar contacts on the interface between the DHp and CA domains with emphasis on the hydrophobic and aromatic residues. Displayed are any changes of contact frequencies between non-polar amino-acid residues (Val, Phe, Ile, Leu, Met, Tyr) within a distance of 0.45 nm. The residues in the DHp domain are on the horizontal axis, and the CA domain is on the vertical, with the ATP-lid highlighted in green. The three different plots are separate maps for three different pairs of conformational states that occur during the activation process: states B–C, C–D and D–A, respectively. Note that this is a non-polar-focused version of Fig. 4 in the main text. The color-coded values range between -1 (for contacts present entirely in the initial state and missing entirely in the final state) and $+1$ (for contacts missing entirely in the initial state and present entirely in the final state). The representative structures of the states I and A have α-helical segments highlighted: α1 (residues 460–476), α2 (502–520) and α3 (575–588) in blue; αA (383–413) and αB (415–445) in green.

Ramachandran plots of the residues of the loop state B

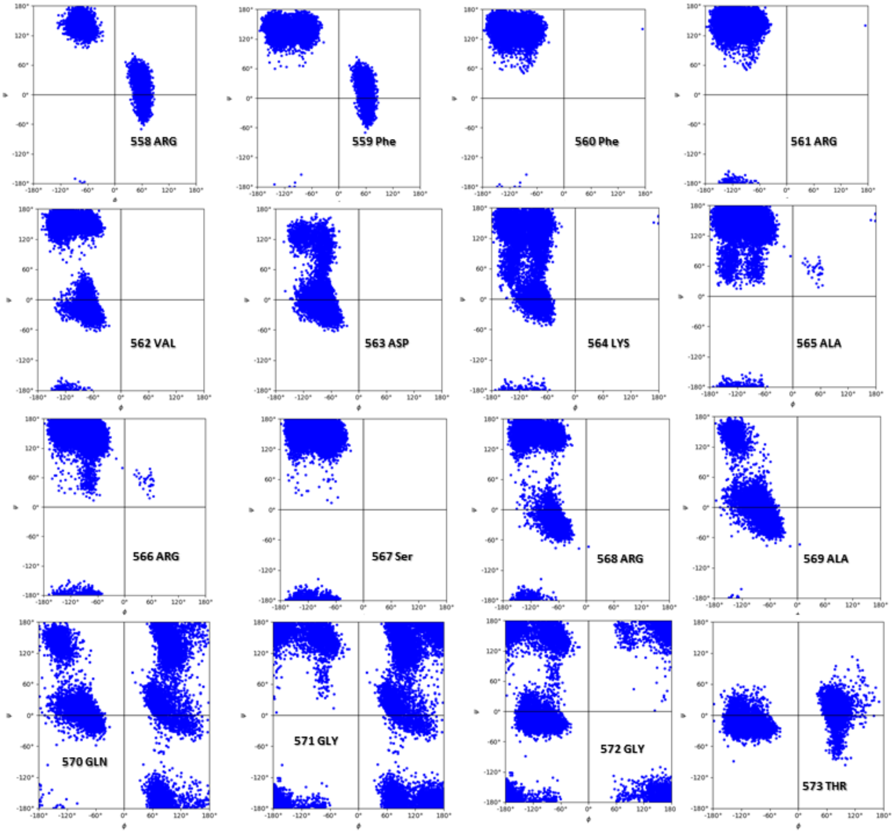

Figure S5

Ramachandran plots of the residues of the loop state C

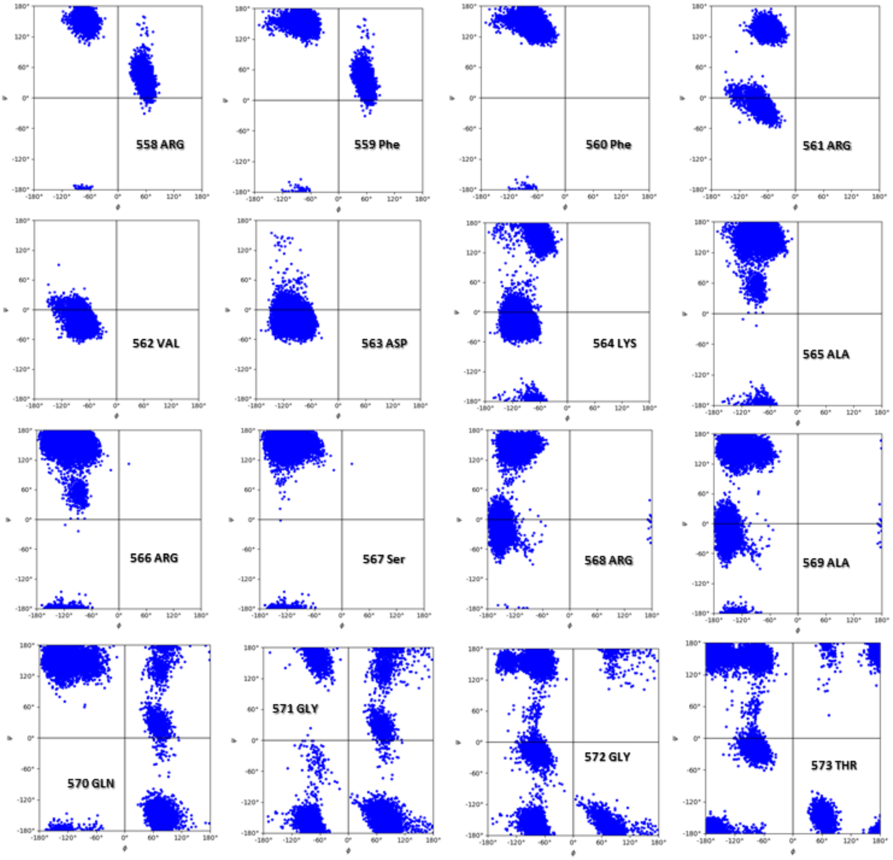

Figure S6

Ramachandran plots of the residues of the loop state D

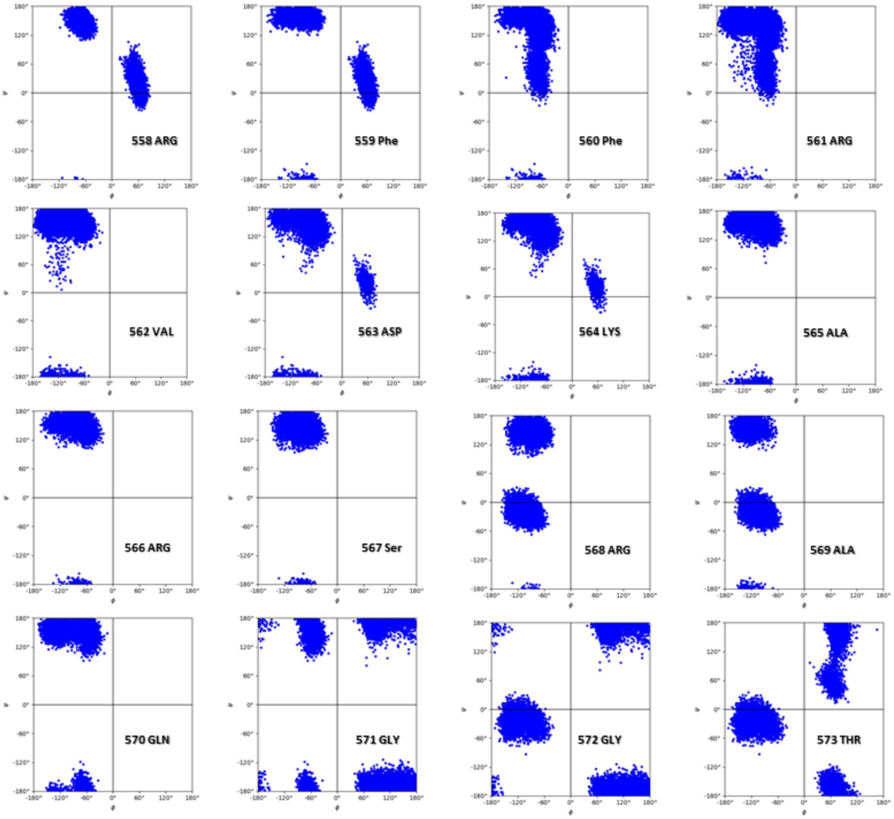

Figure S7

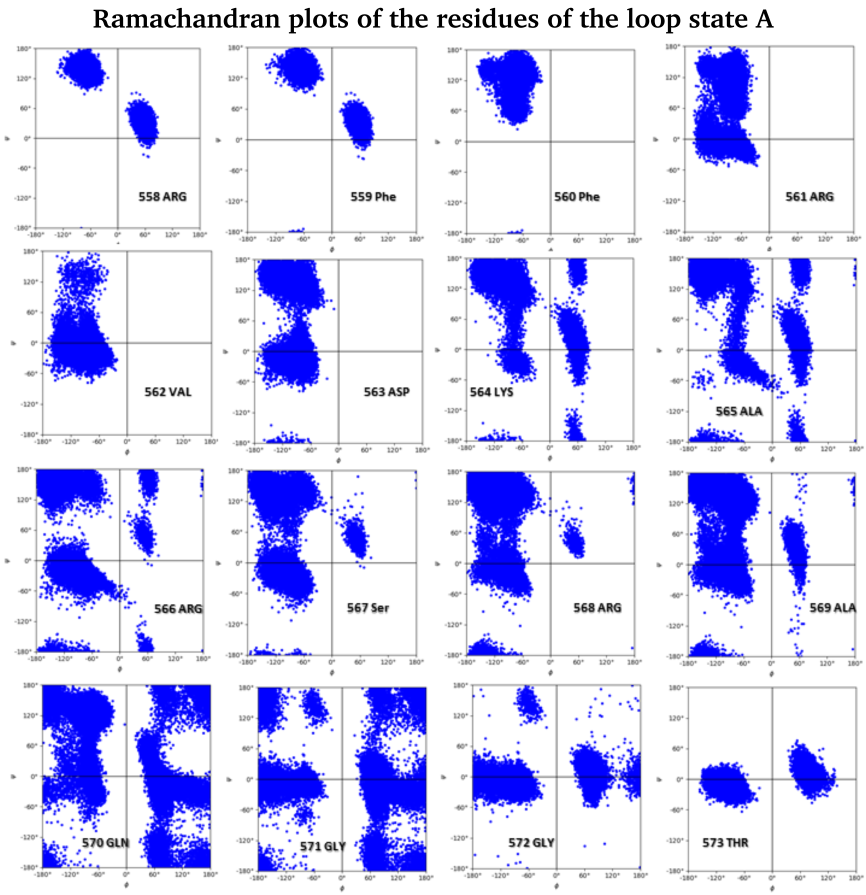
Ramachandran plots of the residues of the loop state A
558 ARG
559 Phe
560 Phe
561 ARG
562 VAL
563 ASP
564 LYS
565 ALA
566 ARG
567 Ser
568 ARG
569 ALA
570 GLN
571 GLY
572 GLY
573 THR

Figure S8

Supplementary Results – Phosphoryl Transfer Reaction

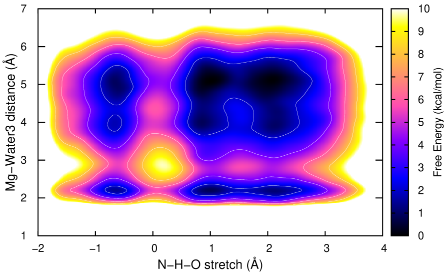

Figure S9 Potentials of the mean force from the 2D metadynamics simulation using as CVs the N–H–O antisymmetric stretch to describe the proton transfer (horizontal axis) and the distance between the Mg^{2+} cation and the most loosely bound water molecule (vertical axis), with the remaining five coordination bonds of Mg^{2+} being restrained.

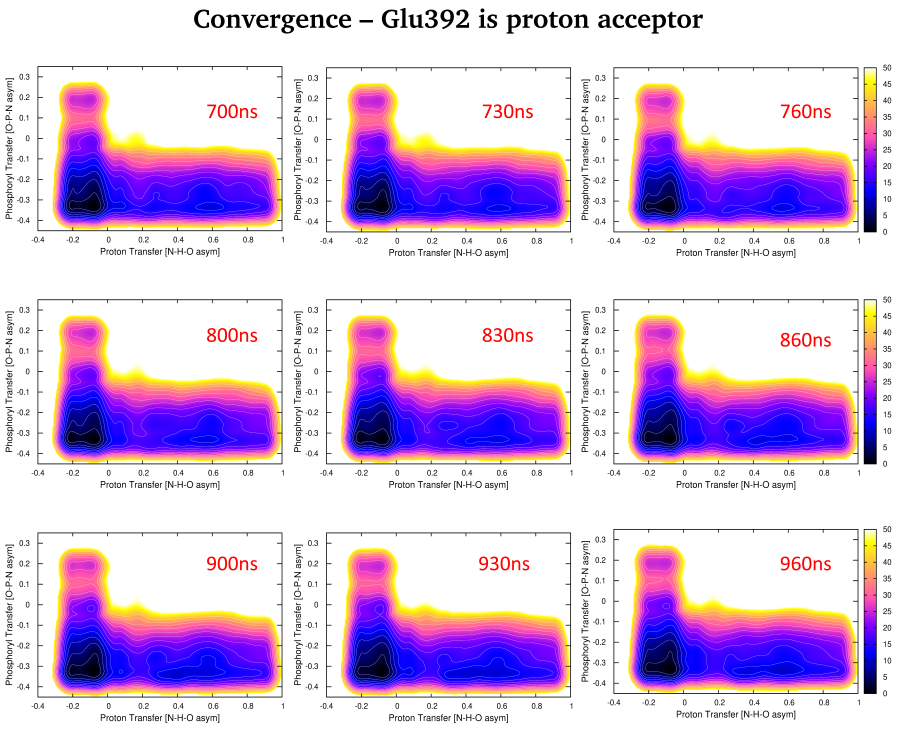

Figure S10 Convergence of the potentials of the mean force in the QM/MM metadynamics simulation of the chemical step of the autophosphorylation, considering the side chain of Glu392 as the proton acceptor. Distances in nm, free energies color-coded in kcal/mol.

Convergence – OH^{-} is proton acceptor

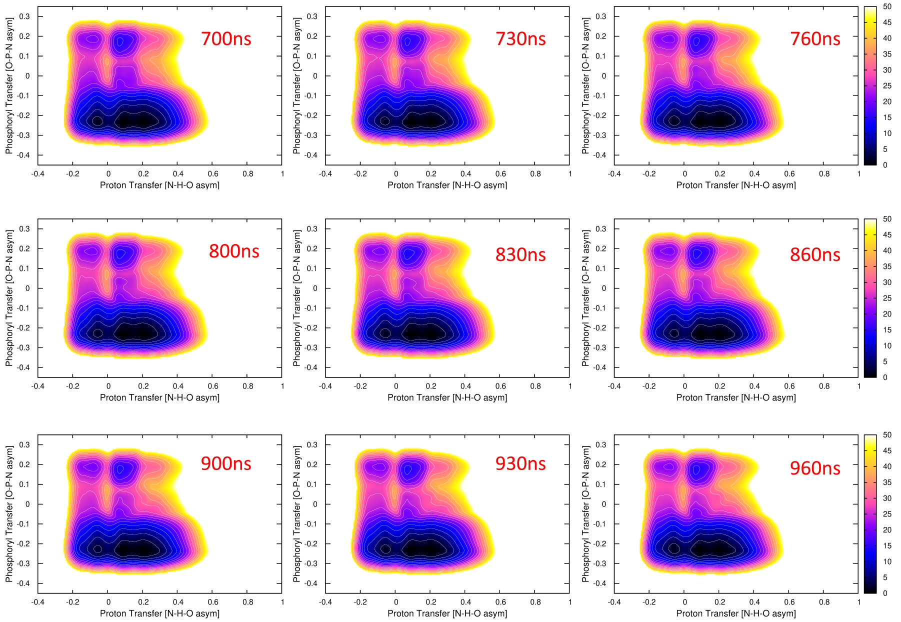

Figure S11 Convergence of the potentials of the mean force in the QM/MM metadynamics simulation of the chemical step of the autophosphorylation, considering a hydroxyl ion as the proton acceptor. Distances in nm, free energies color-coded in kcal/mol.

Convergence of large scale conformational change

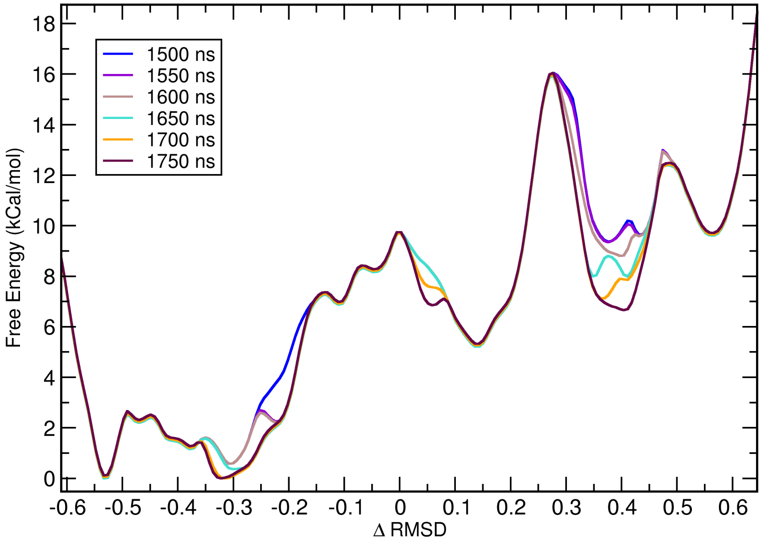

Figure S12 Convergence of the 1D projection of the potentials of the mean force obtained from 2D metadynamics simulation of the conformational change of histidine kinase from inactive to active conformer

Supplementary references

[1] Hess, B., Kutzner, C., van der Spoel, D. & Lindahl, E. GROMACS 4: Algorithms for highly efficient, load-balanced, and scalable molecular simulation. *J. Chem. Theory Comput.* **4**, 435–447 (2008).

[2] Abraham, M. J. *et al.* GROMACS: High performance molecular simulations through multi-level parallelism from laptops to supercomputers. *SoftwareX* **1–2**, 19–25 (2015).

[3] Lindahl, E., Abraham, M., Hess, B. & van der Spoel, D. Gromacs 2020 manual (2020). URL https://doi.org/10.5281/zenodo.3562512.

[4] Bonomi, M., Bussi, G., Camilloni, C., Tribello, G. A. & The PLUMED consortium. Promoting transparency and reproducibility in enhanced molecular simulations. *Nat. Methods* **16**, 670–673 (2019).

[5] Lindorff-Larsen, K. *et al.* Improved side-chain torsion potentials for the Amber ff99SB protein force field. *Proteins Struct. Funct. Bioinformat.* **78**, 1950–1958 (2010).

[6] Jorgensen, W. L., Chandrasekhar, J., Madura, J. D., Impey, R. W. & Klein, M. L. Comparison of simple potential functions for simulating liquid water. *J. Chem. Phys.* **79**, 926–935 (1983).

[7] Darden, T., York, D. & Pedersen, L. Particle–mesh Ewald: An $n \cdot \log(n)$ method for Ewald sums in large systems. *J. Chem. Phys.* **98**, 10089–10092 (1993).

[8] Hess, B., Bekker, H., Berendsen, H. J. C. & Fraaije, J. G. E. M. LINCS: a linear constraint solver for molecular simulations. *J. Comput. Chem.* **18**, 1463–1472 (1997).

[9] Bussi, G., Donadio, D. & Parrinello, M. Canonical sampling through velocity rescaling. *J. Chem. Phys.* **126**, 014101 (2007).

[10] Parrinello, M. & Rahman, A. Polymorphic transitions in single crystals: A new molecular dynamics method. *J. Appl. Phys.* **52**, 7182–7190 (1981).

[11] Yu, L. *et al.* Role of Mg^{2+} ions in protein kinase phosphorylation: insights from molecular dynamics simulations of ATP-kinase complexes. *Mol. Simul.* **37**, 1143–1150 (2011).

[12] Wolanin, P. M., Thomason, P. A. & Stock, J. B. Histidine protein kinases: key signal transducers outside the animal kingdom. *Genome Biol.* **3**, 3013.1 (2002).

[13] Cai, Y. *et al.* Conformational dynamics of the essential sensor histidine kinase WalK. *Acta Crystallographica Section D: Structural Biology* **73**, 793–803 (2017). URL http://scripts.iucr.org/cgi-bin/paper?di5014.

[14] Pettersen, E. F. *et al.* UCSF Chimera – a visualization system for exploratory research and analysis. *Journal of Computational Chemistry* **25**, 1605–1612 (2004). URL https://onlinelibrary.wiley.com/doi/full/10.1002/jcc.20084https://onlinelibrary.wiley.com/doi/abs/10.1002/jcc.20084https://onlinelibrary.wiley.com/doi/10.1002/jcc.20084.

[15] Webb, B. & Sali, A. Comparative protein structure modeling using MODELLER. *Curr. Protoc. Bioinformat.* **54**, 5–6 (2016). URL https://onlinelibrary.wiley.com/doi/full/10.1002/cpbi.3https://onlinelibrary.wiley.com/doi/abs/10.1002/cpbi.3https://currentprotocols.onlinelibrary.wiley.com/doi/10.1002/cpbi.3.

[16] Meagher, K. L., Redman, L. T. & Carlson, H. A. Development of polyphosphate parameters for use with the AMBER force field. *J. Comput. Chem.* **24**, 1016–1025 (2003).

[17] Jorgensen, W. L., Chandrasekhar, J., Madura, J. D., Impey, R. W. & Klein, M. L. Comparison of simple potential functions for simulating liquid water. *J. Chem. Phys.* **79**, 926–935 (1983). URL https://www.scienceopen.

com/document?vid=aa060024-ca0f-4747-973b-3aa32343f353.

[18] Åqvist, J. Ion-water interaction potentials derived from free energy perturbation simulations. *Journal of Physical Chemistry* **94**, 8021–8024 (1990).

[19] Essmann, U. *et al.* A smooth particle–mesh Ewald method. *Journal of Chemical Physics* **103**, 8577 (1998). URL https://aip.scitation.org/doi/abs/10.1063/1.470117.

[20] Cuendet, M. A. & van Gunsteren, W. F. On the calculation of velocity-dependent properties in molecular dynamics simulations using the leapfrog integration algorithm. *Journal of Chemical Physics* **127**, 184102 (2007). URL https://aip.scitation.org/doi/abs/10.1063/1.2779878.

[21] Bussi, G., Donadio, D. & Parrinello, M. Canonical sampling through velocity rescaling. *Journal of Chemical Physics* **126**, 014101 (2007). URL https://aip.scitation.org/doi/abs/10.1063/1.2408420.

[22] Gaus, M., Cui, Q. & Elstner, M. DFTB3: Extension of the self-consistent-charge density-functional tight-binding method (SCC-DFTB). *Journal of Chemical Theory and Computation* **7**, 931–948 (2011). URL https://pubs.acs.org/doi/full/10.1021/ct100684s.

[23] Gaus, M., Goez, A. & Elstner, M. Parametrization and benchmark of DFTB3 for organic molecules. *Journal of Chemical Theory and Computation* **9**, 338–354 (2013). URL https://pubs.acs.org/doi/full/10.1021/ct300849w.

[24] Gaus, M., Lu, X., Elstner, M. & Cui, Q. Parameterization of DFTB3/3OB for sulfur and phosphorus for chemical and biological applications. *Journal of Chemical Theory and Computation* **10**, 1518–1537 (2014). URL https://pubs.acs.org/doi/full/10.1021/ct401002w.

[25] Kubař, T., Welke, K. & Groenhof, G. New QM/MM implementation of the DFTB3 method in the Gromacs package. *Journal of Computational Chemistry* **36**, 1978–1989 (2015). URL https://onlinelibrary.wiley.com/doi/abs/10.1002/jcc.24029. https://onlinelibrary.wiley.com/doi/pdf/10.1002/jcc.24029.

[26] Kubař, T. Gromacs – QM/MM interface for DFTB+. https://github.com/tomaskubar/gromacs-dftbplus (2022). Last accessed 18 March 2022.

[27] Tribello, G. A., Bonomi, M., Branduardi, D., Camilloni, C. & Bussi, G. Plumed 2: New feathers for an old bird. *Computer Physics Communications* **185**, 604–613 (2014).

[28] Hourahine, B. *et al.* DFTB+, a software package for efficient approximate density functional theory based atomistic simulations. *Journal of Chemical Physics* **152**, 124101 (2020). URL https://aip.scitation.org/doi/abs/10.1063/1.5143190.

[29] Kubař, T. DFTB+ – modified QM/MM interface. https://github.com/tomaskubar/dftbplus (2022). Last accessed 18 March 2022.

[30] Raiteri, P., Laio, A., Gervasio, F. L., Micheletti, C. & Parrinello, M. Efficient reconstruction of complex free energy landscapes by multiple walkers metadynamics. *Journal of Physical Chemistry B* **110**, 3533–3539 (2005). URL https://pubs.acs.org/doi/abs/10.1021/jp054359r.

[31] Bussi, G. & Laio, A. Using metadynamics to explore complex free-energy landscapes. *Nature Reviews Physics* **2**, 200–212 (2020). URL https://www.nature.com/articles/s42254-020-0153-0.

[32] Barducci, A., Bussi, G. & Parrinello, M. Well-tempered metadynamics: A smoothly converging and tunable free-energy method. *Physical Review Letters* **100**, 020603 (2008). URL https://journals.aps.org/prl/abstract/10.1103/PhysRevLett.100.020603.
